# The Efficacy of Berberine-Containing Quadruple Therapy on Helicobacter Pylori Eradication in China: A Systematic Review and Meta-Analysis of Randomized Clinical Trials

**DOI:** 10.3389/fphar.2019.01694

**Published:** 2020-02-04

**Authors:** Qian Hu, Ze Peng, Lingli Li, Xin Zou, Lijun Xu, Jing Gong, Ping Yi

**Affiliations:** ^1^Institute of Integrated Traditional Chinese and Western Medicine, Tongji Hospital, Tongji Medical College, Huazhong University of Science and Technology, Wuhan, China; ^2^Department of Integrated Traditional Chinese and Western Medicine, Tongji Hospital, Tongji Medical College, Huazhong University of Science and Technology, Wuhan, China

**Keywords:** berberine, *Helicobacter pylori*, standard triple therapy, eradication rate, systematic review

## Abstract

**Background:**

Berberine-containing quadruple therapy (adding berberine to the standard triple therapy) is being used to treat *Helicobacter pylori* infection, but the effects in randomized controlled trials (RCTs) are still controversial. Therefore, a meta-analysis is needed to estimate the efficacy and safety of berberine-containing quadruple therapy on *Helicobacter pylori* eradication.

**Methods:**

Ten databases were searched to find the available literature. RCTs about the efficacy and safety of berberine-containing quadruple therapy on *Helicobacter pylori* eradication were included. The data of *Helicobacter pylori* eradication rate, peptic ulcer healing rate, relieving rate of clinical symptoms and adverse events were extracted to appraise the net change with a fixed or randomized effect model.

**Results:**

A total of 13 articles were included in the analysis. Pooled results showed that the addition of berberine in standard triple therapy significantly improved *Helicobacter pylori* eradication rate (RR 1.22; 95% CI 1.16 to 1.27; I^2^ = 12%), increased the peptic ulcer healing rate (RR 1.15; 95% CI 1.10 to 1.19; I^2^ = 44%), relieved the clinical symptoms (RR 1.11; 95% CI 1.06 to 1.17; I^2^ = 44%) and reduced the incidence of side events (RR 0.65; 95% CI 0.53 to 0.80; I^2^ = 58%) comparing to the standard triple therapy.

**Conclusions:**

The analysis showed that the addition of berberine in standard triple therapy could improve *Helicobacter pylori* eradication rate and clinical symptom remission rate, accelerate ulcer healing, and reduce adverse events, which is very beneficial to clinical work in China.

## Introduction

*Helicobacter pylori (H. pylori)* infection is highly prevalent worldwide. In Northern Europe and North America, approximately one-third of adults are infected. Worse still, in South and East Europe, South America, and Asia, the prevalence of *H. pylori* is higher than 50% (Eusebi and Zagari, 2014; Xu et al., 2014). *H. pylori* infection is highly associated with the occurrence of gastrointestinal diseases, including gastric inflammation, peptic ulcers, gastric cancer, and gastric mucosa-associated lymphoid tissue lymphoma (Yang and Lu, 2014; Debraekeleer, 2018). In China, the infection rate of *H. pylori* is 40%–60% (Chen et al., 2019). In recent years, in addition to its role in gastrointestinal diseases, several nongastric issues have been found to have a close relationship with *H. pylori* infection (Adachi et al., 2018; Yong and Upala, 2018; Lee et al., 2018). *H. pylori* treatment relies on a combination of antimicrobial agents, such as amoxicillin, clarithromycin, metronidazole, and antisecretory agents, such as proton pump inhibitors (PPIs) (Yang and Lu, 2014). A standard triple therapy consisting of a PPI and two antibiotics (clarithromycin and amoxicillin) has been widely used in China since 2011. With the resistance of *H. pylori* to antibiotics increasing, the efficacy of triple therapy is declining. Bismuth-containing quadruple therapy was proposed in China in 2012 (Liu et al., 2012). Unfortunately, a questionnaire survey among digestive doctors in 100 hospitals across the country showed that the application rate of bismuth-containing quadruple therapy was only 33% in China until 2017 (Xie and Lv, 2017). What’s the reason? Adding bismuth to triple therapy reduced the compliance of patients because of its high frequency of adverse events, such as vomiting, abdominal pain, black tongue, and diarrhea (Graham and Lee, 2015; Kahramanoğlu et al., 2017). More importantly, bismuth has been substituted by berberine, a traditional Chinese medicine (TCM), for the eradication of *H. pylori* in China.

Berberine is a kind of isoquinoline alkaloid isolated from TCM Rhizoma coptidis and cypress. As an “Eastern antibiotic” in TCM, berberine has been used to treat diarrhea for a thousand years (Zhang, 1992). Recently, many experiments have proved that berberine has anti-*H. pylori* ability *in vivo* and *in vitro* (Huang et al., 2015; Wu et al., 2018). Berberine can effectively suppress multiresistant strains of *H. pylori*, and the minimum *H. pylori* inhibitory concentration of amoxicillin and tetracycline was lowered after the berberine intervention *in vitro* (Huang et al., 2015). Berberine can also suppress the expression of proinflammatory genes and upregulate antiinflammatory gene expression in *H. pylori*-infected mice (Wu et al., 2018). With the efficacy of the standard triple therapy for *H. pylori* declining, the anti-*H. pylori* property of berberine has received more attention. In China, an increasing number of randomized controlled clinical trials confirmed the efficacy of berberine combined with standard triple therapy on *H.pylori* eradication. A recent open-label randomized phase IV trial showed that both berberine-containing quadruple therapy and bismuth-containing quadruple therapy achieved the recommended efficacy and can be recommended as the first-line treatment for *H. pylori* eradication in Xi’an, China (Zhang et al., 2017). Therefore, based on the extensive collection of literature, we designed this randomized controlled trial to assess the efficacy and safety of the addition of berberine in standard triple therapy on *H. pylori* eradication.

## Materials and Methods

### Methods

We conducted and reported this review according to the Preferred Reporting Items for Systematic Reviews and Meta-Analyses (PRISMA) Statement protocol (Moher et al., 2015).

### Search Strategy

To determine the effect of berberine on *H. pylori* eradication, PubMed, EMBASE, Web of Science, the Cochrane Library, the Chinese Biomedical Literature Database (CBM), the Wan Fang Medical Database, the China National Knowledge Internet (CNKI), the China Academic Journal Network Publishing Database (CAJD), the Chinese Science Citation Database (CSCD), and the China Science and Technology Journal Database (CSTJ) were searched. All the above databases were searched from the available date of inception until the latest issue (January 2018). No language restriction was used.

Search strategies were as follows: for English databases, we use text terms such as berberine and *Helicobacter pylori* or berberine and *H. pylori*; for Chinese databases, we use text terms such as “Huang Lian Su” or “Xiao Bo Jian” and “You Men Luo Xuan Gan Jun” or “You Men Luo Gan Jun.” Huang Lian Su and Xiao Bo Jian are the alternative names for berberine in Chinese; You Men Luo Xuan Gan Jun and You Men Luo Gan Jun are the alternative names of *H. pylori* in Chinese. A filter for clinical trials was applied. References were also searched to retrieve the related literature. Through reading the title and summary, the preliminary screening was completed. Then full texts of the applicable studies were downloaded and screened.

### Selection Criteria

The inclusion criteria of randomized clinical trials (RCTs) were as follows. (1) Patients with *H. pylori* infections that were positive, preexisted or newly diagnosed (either urea breath test [UBT] or rapid urease test [RUT]) were included. (2) The standard triple therapy (two antibiotics and one PPI) was chosen as a control group; the berberine-containing quadruple therapy (adding berberine to the standard triple therapy) was chosen as the experimental group, which means that berberine added to the treatment group was the only treatment difference between the two groups. (3) The data of outcomes (*H. pylori* eradication rate, improvement of ulcers, relieving rate of clinical symptoms and adverse events) were countable.

The excluded criteria were as follows: (1) Case reports, reviews, animal or cell studies, and studies without a control group. (2) Berberine combined with other drugs served as a control group, or berberine was substituted for an antibiotic in the experiment group. (3) The control group did not adapt to the standard triple therapy. (4) The data of outcomes were not accessible.

### Data Extraction

Two reviewers (Qian Hu and Ze Peng) independently extracted the information. They extracted data concerning details of the author, publication year, diagnosis, diagnostic method, age, the number of participants, characteristics for both intervention and control group (treatment protocols, drug name), treatment duration, and outcomes. The primary outcomes consisted of *H. pylori* eradication rate, improvement of ulcers, relieving rate of clinical symptoms and adverse events. Where outcomes were ambiguous or missing in an article, the decision to retrieve from that article was resolved by consensus.

### Methodological Quality

Jadad scores were used to assess the quality of each included study independently. The Jadad score is usually evaluated through three main parts as described below: the method of randomization (0–2 points), blind method (0–2 points), and description of withdrawals and dropouts (0–1 point). With a total score of five, studies with Jadad scores higher than 3 were regarded as high quality; otherwise, it was considered low quality. All of the included studies had a high Jadad score in this meta-analysis.

### Data Synthesis and Analysis

Review Manager meta-analysis software 5.3 and Stata 12.0 software were used to summarize the effect of berberine. We calculated the risk ratio (RR) and the 95% confidence interval (CI) for count data. The heterogeneity was evaluated with the chi square test, df test, Tau test, and the Higgins I^2^ test. The heterogeneity test was conducted among the studies, with P < 0.05 being the test level. When P > 0.05, there was no significant heterogeneity among the studies, and the fixed-effect model was selected for analysis; otherwise, the random effect model was used for the combined analysis. The overall effect was tested by using the Z score with significance set at P < 0.05. Egger’s tests were carried out using Stata 12.0 software to determine the publication biases.

## Results

### Study Inclusions

**Figure 1:**
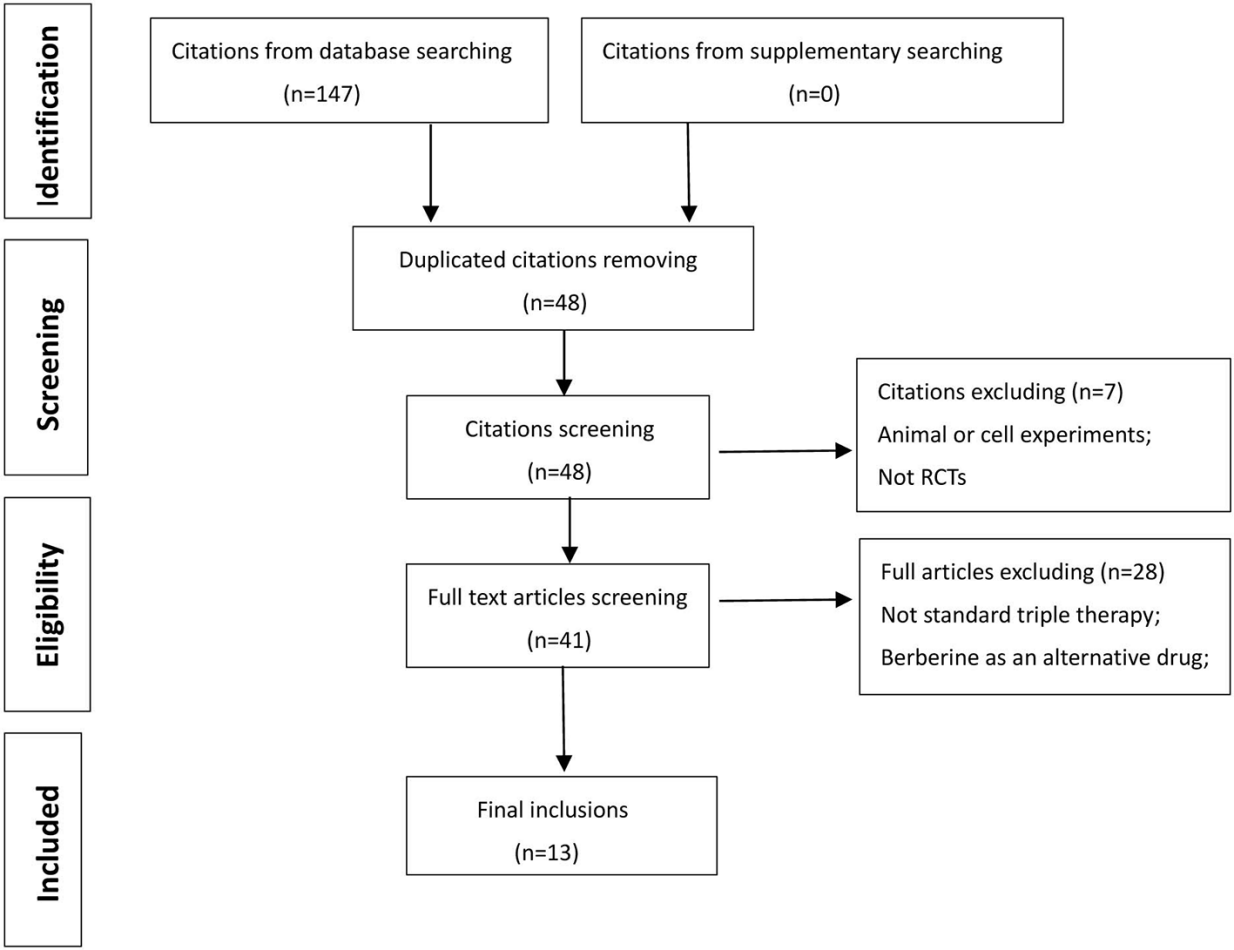
Flow Chart of study selection.

As illustrated in **Figure 1**, 147 studies were retrieved from the databases. With the removal of duplicates, the titles and abstracts of 48 studies have remained. The remaining studies were further carefully searched, and seven studies were eliminated due to animal or cell experiments (n = 3) and nonrandomized clinical trials (n = 4). Then, 28 records were excluded because of no standard triple therapy (n = 10) in the control group or berberine as an alternative drug (n = 18) in the experiment group. Finally, 13 articles were included in our meta-analysis. (Ma et al., 2011; Chen et al., 2013; Dong and Dai., 2013; Lu et al., 2013; Qi and Xiao, 2013; Si and Hu, 2013; Zhou et al., 2013; Zhang GF, 2014; Zhang XX, 2014; Hu, 2015; Ma, 2016; Huang et al., 2017; Luo et al., 2017).

### Characteristics of the Studies

Thirteen RCTs published as full text between 2011 and 2017 met our inclusion criteria. All RCTs originated from China and were published in Chinese. The details of the trials are listed in **Table 1**. Together, those trials included 2,111 patients. In the studies included, there were no significant differences in the general data (age, gender, duration of illness) between the control group and the experimental group. All subjects were positive for *H. pylori*. The control group received the standard triple therapy. Based on the standard triple therapy, berberine was added separately to the experiment group. The efficacy of adding berberine to triple therapy for *H. pylori* eradication was studied. Additionally, ten of them gave the outcomes about the healing of ulcers using a gastroscope, and five of them described the improvement of clinical symptoms after treatment. All studies recorded adverse reactions.

**Table 1:** Characteristics of included trials (two-armed parallel group).

Author and year	Diagnosis	Number of patients		Average Age or range	Intervention	Control	Duration	Outcome	Dose	Method (A or B)
		Experiment	control							
Ma et al., 2011	PU/CG	54	42	40.94 ± 15.64	RAB,AMO,CLA,BBR	RAB,AMO,CLA	7d	Hp, PU, AE	120mg	A
Si and Hu, 2013	PU, Hp^+^	45	45	43.5 ± 3.7	OME,AMO,CLA,BBR	OME,AMO,CLA	14d	Hp, PU, AE	400mg	A B
Qi and Xiao, 2013	Hp^+^	210	210	44.2 ± 2.5	RAB,AMO,CLA,BBR	RAB,AMO,CLA	7d	Hp, AE	300mg	A
Lu et al., 2013	Hp^+^	84	78	40.87 ± 14.64	RAB,AMO,CLA,BBR	RAB,AMO,CLA	7d	Hp, PU, AE	120mg	A
Zhou et al., 2013	Hp^+^, T2DM	50	50	30–75	OME,AMO,CLA,BBR	OME,AMO,CLA	14d	Hp, PU, AE, FBG	300mg	B
Chen et al., 2013	Hp^+^	140	125	55 ± 26	OME,AMO,CLA,BBR	OME,AMO,CLA	14d	Hp, AE, CS	300mg	A
Dong and Dai, 2013	Hp^+^	50	50	39.8 ± 15.1	RAB,AMO,CLA,BBR	RAB,AMO,CLA	7d	Hp, PU, AE,CS	120mg	B
Zhang, 2014	Hp^+^	100	100	43.3 ± 14.4	RAB,AMO,CLA,BBR	RAB,AMO,CLA	14d	Hp, PU, AE	120mg	B
Zhang, 2014	PU	50	50	43.5 ± 3.7	OME,AMO,CLA,BBR	OME,AMO,CLA	14d	Hp, PU, AE,CS	500mg	B
Hu, 2015	Hp^+^	53	53	49.7 ± 10.6	OME,AMO,CLA,BBR	OME,AMO,CLA	14d	Hp, PU, AE	500mg	A
Ma, 2016	Hp^+^	55	55	28–58	RAB,AMO,CLA,BBR	RAB,AMO,CLA	7d	Hp, AE, CS	120mg	B
Huang et al., 2017	PU, Hp^+^	100	100	46.2 ± 11.0	OME,AMO,CLA,BBR	OME,AMO,CLA	14d	Hp, PU, AE	300mg	A B
Luo et al., 2017	Hp^+^, PU,T2DM	81	81	27–57	OME,AMO,CLA,BBR	OME,AMO,CLA	14d	Hp, PUAE, FBG,CS	120mg	B

RAB, rabeprazole; OME, omeprazole; AMO, amoxicillin; CLA, clarithromycin; BBR, berberine; PU, peptic ulcer; CG, chronic gastritis; Hp^+^:helicobater pylori positive; AE, adverse events; CS, clinical symptom; T2DM, type 2 diabetes; FBG, fasting blood glucose; Dose, the dose of Berberine; Method, A-uera breath test; B-Rapid urease test.

Two kinds of triple therapies were used in all the included studies: omeprazole, clarithromycin, and amoxicillin (n=7) and rabeprazole, clarithromycin, and amoxicillin (n = 6). Concerning treatment time, five articles were for 7 days, and eight articles were for 14 days. All experimental groups received additional oral berberine tablets three times a day. The dose of berberine varied in all included studies: 120 mg in six papers, 300 mg in four papers, 400 mg in one paper, and 500 mg in two papers. There are seven studies in which the dose of berberine was greater than or equal to 300 mg. At present, two methods were used to determine the Hp infection: the UBT and the RUT. In the included literature, there are five studies using UBT as the criterion, six papers as the standard of RUT, and two papers combined both UBT and RUT. The correct detection time of Hp eradication is 28 days after treatment, eight cases stated 4 weeks after the end of treatment, and five cases did not mention the specific time.

### Quality of Included Studies

Based on Jadad scores, all studies were deemed high quality. Eight studies scored three points, and the rest scored four points. These studies reported the randomized method, blind and withdrawal/dropout. The average score of 3.38 indicated the risk of bias (shown in **Supplementary Table 1**).

### *H. pylori* Eradication Rate

**Figure 2:**
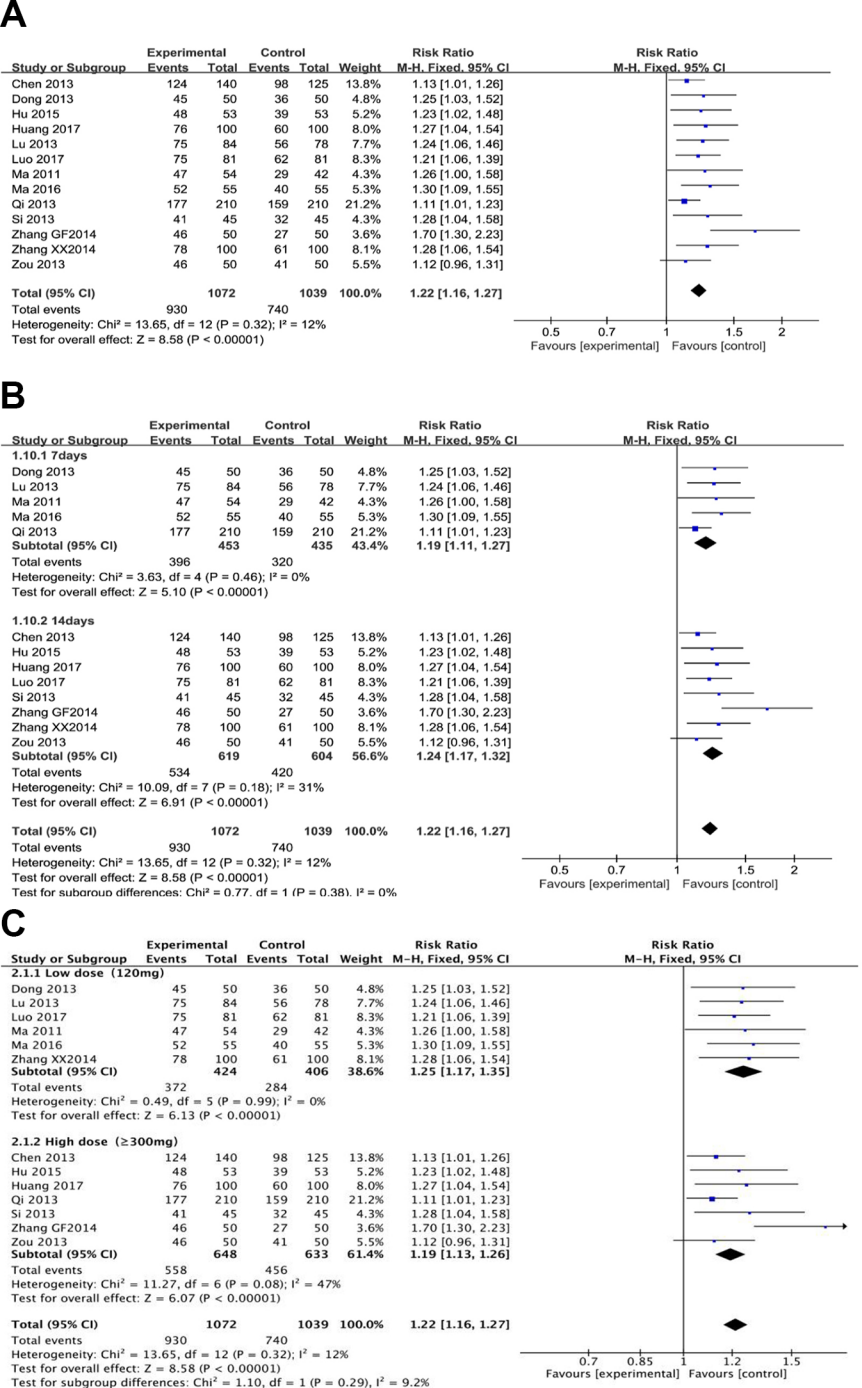
The pooled effects of berberine-containing quadruple therapy on *H.pylori* eradication rate **(A)**. Forest plot comparing berberine-containing quadruple treatment with the standard triple therapy; **(B)**. Subgroup analysis about the treatment duration; **(C)**. Subgroup analysis about the dose of berberine.

Thirteen trials (involving 2,111 patients) evaluated the *H. pylori* eradication effect of berberine with the standard triple therapy versus the standard triple therapy. The heterogeneity result showed that the included studies had clinical and statistical homogeneity, so a fixed-effect model was chosen. As shown in **Figure 2A**, the berberine-containing group had a higher Hp eradication rate than the control group (RR 1.22; 95% CI 1.16 to 1.27; I^2^ = 12%). The results of the meta-regression showed that there was no difference regarding the publication year, the method, the PPI type, the total number of participants, the dose of berberine, and the time of treatment duration (shown in **Supplementary Figure 1**). The subgroup analyses regarding the time of treatment duration suggested no significance (**Figure 2B**). In the subgroup concerning the dose of berberine, we combined 300, 400, and 500 mg into one group and grouping the dose as low dose (120 mg) and high dose (≥300 mg). The result indicated that there is no significant difference between low dose group and high dose group for the efficiency of *H. pylori* eradication, but more trials needed to be performed (**Figure 2C**).

### Peptic Ulcer Healing Rate

**Figure 3:**
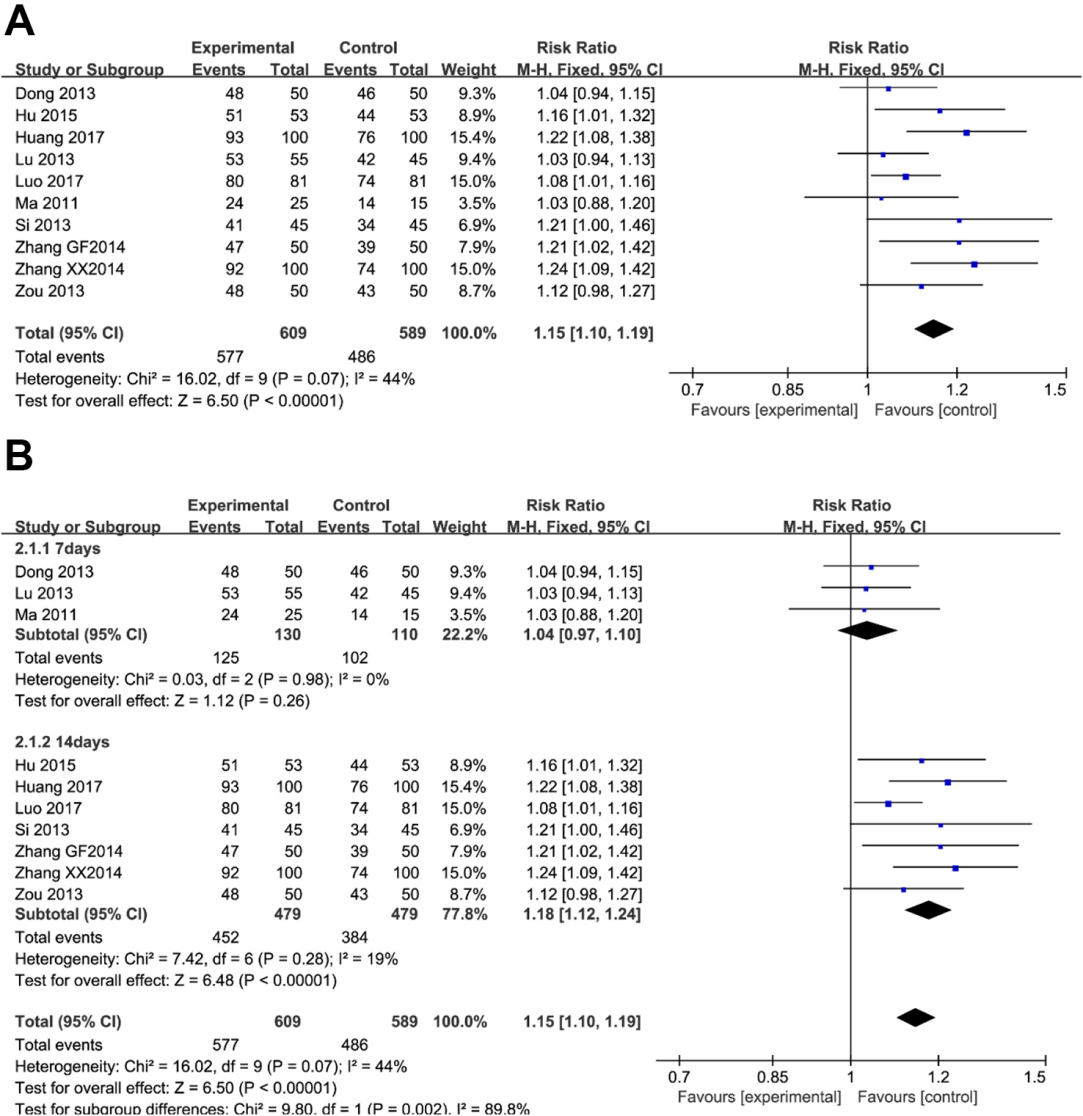
The pooled effects of berberine-containing quadruple therapy on peptic ulcer healing rate. **(A)** Forest plot comparing berberine-containing quadruple treatment with the standard triple therapy. **(B)** Subgroup analysis about the treatment duration.

Ten trials (involving 1,198 patients) recorded conditions of the ulcer lesions after treatment. There was no clinically and statistically significant heterogeneity in these trials. When red or white scars formed, ulcers completely healed, or ulcer lesions shrunk below 50%, and the treatment group was judged to be effective. As shown in **Figure 3A**, there were more effective cases in the berberine-containing group (577/609) than in the control group (486/589) (RR 1.15; 95% CI 1.10 to 1.19; I^2^^=^ 44%). The results of the meta-regression showed that there was no difference regarding the publication year, the method, the PPI type, the total number of participants, the dose of berberine, and the time of treatment duration (shown in **Supplementary Figure 2**). There was no significant change in sensitivity analyses. However, in the subgroup concerning the time of treatment duration, the result indicated a significant difference (**Figure 3B**).

### Relieving Rate of Clinical Symptoms

**Figure 4:**
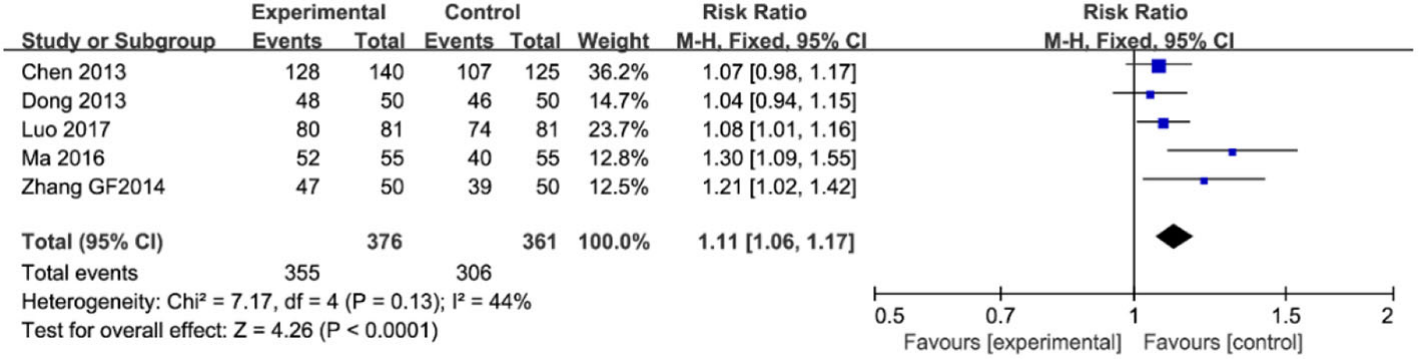
The pooled effects of berberine-containing quadruple therapy on relieving rate of clinical symptoms. Forest plot comparing berberine-containing quadruple treatment with the standard triple therapy.

As illustrated in **Figure 4**, five trials (involving 737 patients) described the improvement of clinical symptoms of berberine-containing quadruple therapy. The included literature classified the improvement of clinical symptoms into complete remission, marked effect, effectivity, and inefficiency. Considering that meta-analysis only performs binary variable analysis on the count data (if valid and invalid), we combined the first three outcomes with the result of clinical improvement. The results showed that the clinical improvement of the treatment group was better than that of the control group (RR 1.11; 95% CI 1.06 to 1.17; I^2^ = 44%). The results of meta-regression showed that there were no differences regarding the PPI type, the dose of berberine, and the time of treatment duration. The subgroup analyses and sensitivity analysis demonstrated no significant change (shown in **Supplementary Figure 3**).

### Adverse Effect

**Figure 5:**
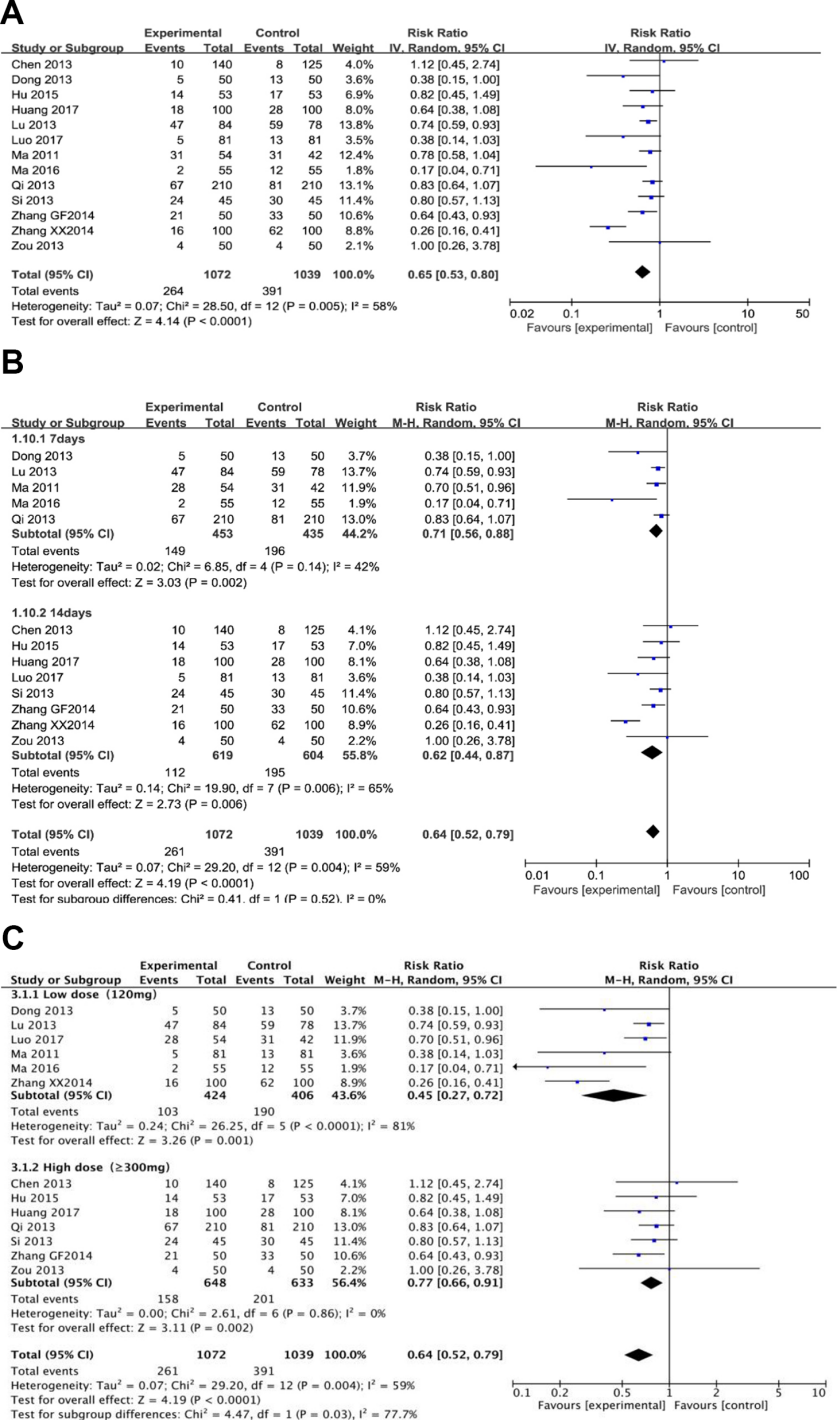
The pooled effects of berberine-containing quadruple therapy on adverse effect. **(A)**. Forest plot comparing berberine-containing quadruple treatment with the standard triple therapy; **(B)**. Subgroup analysis about the treatment duration; **(C)**. Subgroup analysis about the dose of berberine.

Thirteen trials (involving 2,111 patients) evaluated the adverse events in the berberine-containing group compared with the control group. Side events were mainly manifested as gastrointestinal reactions, such as nausea, vomiting, abdominal distension, diarrhea, and anorexia. Heterogeneity tests (I^2^^=^ 58%) suggested that there was considerable heterogeneity, so we turned to a random effect model. As shown in **Figure 5A**, the experimental group significantly reduced the incidence of side events (RR 0.65; 95% CI 0.53 to 0.80; I^2^^=^ 58%). The results of the meta-regression showed that there was no difference regarding the publication year, the method, the PPI type, the total number of participants, the dose of berberine, and the time of treatment duration (shown in **Supplementary Figure 4**). The subgroup analyses regarding the time of treatment duration suggested no significance (**Figure 5B**). As shown in **Figure 5C**, the incidence of adverse events was significantly reduced in the subgroup (low dose = 120 mg) (RR 0.45; 95% CI 0.27 to 0.72).

### Publication Bias

**Figure 6:**
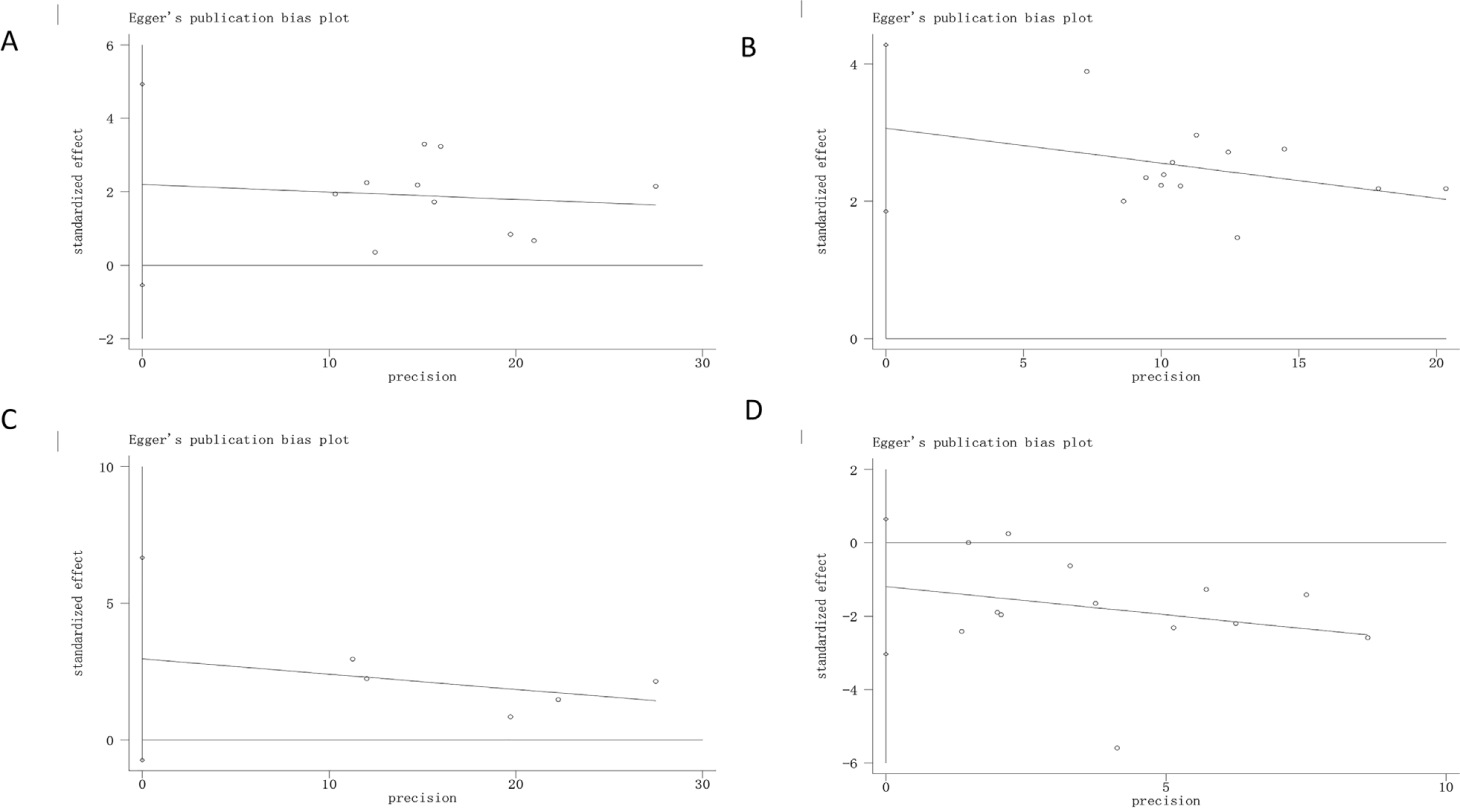
Egger’s regression analyses for publilcation bias. **(A)** *H. pylori* eradication rate; **(B)** Peptic ulcer healilng rate; **(C)**. Relieving rate of clinical symptoms; **(D)** Adverse effect. “0” is a size graph symbol for weights of every included study.

Publication bias was judged by Egger’s tests in Stata 12.0. In Egger’s tests, *p* > 0.05 was considered as no publication bias. The results showed that there was no bias in the analyses of adverse effects (p = 0.178), improvement of ulcers (p = 0.102) and relief rate of clinical symptoms (p = 0.084), except for *H. pylori* eradication rate (p = 0.000) (**Figure 6**). Even though the overall effect of the *H. pylori* eradication rate was significant, Egger’s tests suggested the possibility of publication, and the pooled results need further verification.

## Discussion

In the past decade, the standard triple therapy consisting of a PPI and two antibiotics (clarithromycin and amoxicillin) was the most commonly used for clinical treatment for *H. pylori* infection in China. However, many clinical studies have shown that the initial Hp eradication rate of standard triple therapy is gradually decreasing, and the resistance of *H. pylori* to antibiotics is the main reason for its eradication failure (Papastergiou and Georgopoulos, 2014; Yang and Lu, 2014; Savoldi et al., 2018). Since then, bismuth combined with triple therapy has been proposed in China in 2012, but the addition of bismuth reduced patient compliance because of its high frequency of adverse events, such as vomiting, abdominal pain, black tongue, and diarrhea (Liu et al., 2012). The limited effectiveness of standard triple therapy and the side effect of bismuth-containing quadruple therapy have forced researchers to consider alternative strategies to eliminate *H. pylori* infection. Many new strategies have been proposed, such as supplements of probiotics and TCM. Studies of TCM on *H. pylori* have shown that some herbs such as *R. coptidis, C. phellodendri*, and berberine can inhibit *H. pylori* (Li et al., 2016). Many clinical trials have also shown that berberine, an antimicrobial, antidiabetic, anti-inflammatory, and antitumor drug can be used to treat *H. pylori*-induced chronic gastritis *in vitro* and *in vivo* (Chang et al., 2011; Lin et al., 2015; Tan et al., 2017; Wu et al., 2018). Furthermore, an open-label randomized phase IV trial showed that both bismuth-containing and berberine-containing quadruple therapy achieved the recommended efficacy and can be recommended as the first-line treatment for *H. pylori* eradication in Xi’an, China (Zhang et al., 2017). Although many facts have shown that the addition of berberine in standard triple therapy can improve the eradication rate of *H. pylori*, reduce adverse events, and decrease costs (Li, 2011; Lu, 2018), the effects of berberine-containing quadruple therapy of randomized controlled trials (RCTs) for *H. pylori* eradication are still controversial. Therefore, we conducted a systemic review and meta-analysis on the efficacy and safety of the addition of berberine in standard triple therapy on *H. pylori* eradication.

Our review is a systematic review about the efficacy of the addition of berberine in standard triple therapy on H. *pylori* eradication in China. Our results indicated that berberine-containing quadruple therapy was more effective in terms of *H. pylori* eradication, peptic ulcer healing, and improvement of clinical symptoms. Compared with standard triple therapy (rabeprazole or omeprazole, amoxicillin, and clarithromycin), the addition of berberine in standard triple therapy significantly improved the Hp eradication rate. Although the Hp eradication rates were determined by different methods, either by histological RUT or the ^13^C/^14^C UBT, or a combination of both, all of them can effectively determine the eradication rate of Hp. The dose of berberine varied in these included studies, with most studies using 120 or 300 mg each time, one study using 400 mg each, and two studies using 500 mg each time, three times a day. In our subgroup analyses, we combined 300, 400, and 500 mg into one group and grouping the dose as low dose (120 mg) and high dose (≥300 mg). The result showed that the H. pylori eradication rate didn’t increase with higher dose, but the dose of 120 mg had minimal side events.

*H. pylori* infection plays an important role in the pathogenesis of peptic ulcer disease (PUD). Bacterial adhesins and gastric inflammation are considered to be the main causes of peptic ulcers (Miftahussurur, 2015). A meta-analysis reported that PUD is more common in *H. pylori-*positive patients, and *H. pylori* eradication therapy can reduce PUD incidence (Tang et al., 2012). In 1989, berberine was found to inhibit the formation of an ethanol-induced ulcer, aspirin-induced ulcer, and *H. pylori*-infected ulcer (Uchiyama and Kamikawa, 1989). Recent studies have reported that berberine can protect the gastric mucosa from being damaged by ethanol and attenuate intestinal mucosal barrier dysfunction in type 2 diabetic rats (Pan et al., 2005; Gong et al., 2017). Based on these findings, we further explored the efficiency of berberine-containing quadruple therapy for peptic ulcers. In these included studies, 10 studies reported the healing condition of ulcers. The outcomes of peptic ulcer healing varied. Four of the 10 included articles judged the healing of ulcers where ulcer lesions were significantly improved or ulcers were completely healed (red or white scars formed) under gastroscopy. The remaining six articles judged healing as scar formation or ulcer lesions shrink below 50%. Our review showed that Hp-infected ulcer lesions were significantly improved by adding berberine to the standard triple therapy compared with standard triple therapy. This phenomenon illustrated that berberine can help increase the eradication rate of *H. pylori*.

The clinical symptoms of *H. pylori* infection are sometimes not obvious, sometimes acid reflux, heartburn, and stomachache. This is mainly due to *H. pylori*-induced secretion of gastrin. In the included studies, five articles reported improvement in clinical symptoms. Next, we investigated whether berberine-containing quadruple therapy can improve clinical symptoms. The results showed that the addition of berberine in standard triple therapy could significantly improve clinical symptoms and reduce the frequency compared with the standard triple therapy. The possible reason was that berberine protects the gastrointestinal mucosa and kills *H. pylori* to reduce gastrin release.

In addition, the berberine-containing quadruple therapy in our review appeared to be safe. The adverse reactions were commonly gastrointestinal discomfort, including nausea, vomiting, bloating, diarrhea, anorexia, and few mentioned the abnormal taste and liver function. As shown in the analysis, the adverse event rates were 24.6% (264/1072) and 37.6% (391/1039) in the experimental group and the control group, respectively. However, there was greater heterogeneity in the included studies. The results of the meta-regression showed that there was no difference regarding the publication year, the method, the PPI type, the total number of participants, the dose of berberine, and the time of treatment duration. Although the time of treatment duration was different in different studies, varying from 7 days to 10 days to 14 days, Chen’s study performed a sequential experiment, and the results showed that the *H. pylori* eradication rate and clinical symptom remission rate of 7 days were lowest, and the adverse reactions were fewest (Chen et al., 2013). The *H. pylori* eradication rate and clinical symptom remission rate of 14 days were the highest, and the incidence of adverse reactions increased accordingly. The 10 day treatment had a good *H. pylori* eradication rate and clinical symptom relief rate, and the incidence of adverse reactions was relatively low. There was no significant difference between the 10 days and 14 days in the *H. pylori* eradication rate, so the 10 day treatment course was the best in terms of comprehensive efficacy and safety (Chen et al., 2013). However, in the fifth national consensus report on the treatment of *H. pylori* infection (in China, 2016), treatment for 14 days is recommended (Liu et al., 2018).

### Limitations

There are also some limitations to our study. First, all trials included were conducted in mainland China, and it is still questionable whether the results are applicable in other areas due to genetics and region differences. *Helicobacter pylori* eradication should be assessed on the 28th day after the end of treatment with 13^C^/14^C^-UBT and/or RUT. Ten out of 13 studies confirmed the test time, but three studies did not mention the detection time. Therefore, the literature included in this study is not rigorous enough. These results require further study. Third, in the included studies, the dosage of berberine and treatment duration were not completely consistent. Finally, there are some limitations in the included literature and methodology in this systematic evaluation. The Jadad score of the included studies is generally high, and Egger’s tests illustrated that there was no publication bias in these studies except for the Hp eradication rate. Bias may result from poor design of clinical research such as randomized, controlled, and blind methods and small sample sizes. Most of the included studies were too simple in the description of the random method, and the courses of treatment in these studies were inconsistent. The baseline comparability and loss of visit/withdrawal were not fully reported, and there were differences in the dosage and measurement methods, all of which could have an impact on the results and result in low test efficacy. Therefore, the effect of adding berberine to the standard triple therapy on *H. pylori* should be carefully interpreted based on substantial methodological and clinical research design.

### Conclusions

The analysis showed that the addition of berberine in standard triple therapy could improve the *H. pylori* eradication rate and clinical symptom remission rate, accelerate ulcer healing and reduce adverse events compared to the standard triple therapy, which provided a new method for the eradication of *H. pylori* and is very beneficial to clinical work in China.

## Author Contributions

PY designed the study. QH and ZP conducted the experiments and wrote the manuscript. LL, XZ, LX and JG revised the manuscript. All authors approved the final version to be published.

## Funding

This article was supported by the National Natural Science Foundation of China (No.81673757).

## Conflict of Interest

The authors declare that the research was conducted in the absence of any commercial or financial relationships that could be construed as a potential conflict of interest.
