# Supplementary material for: The Efficacy of Berberine-Containing Quadruple Therapy on Helicobacter Pylori Eradication in China: A Systematic Review and Meta-Analysis of Randomized Clinical Trials
Source: Front Pharmacol. 2020 Feb 4;10:1694. doi: 10.3389/fphar.2019.01694 (PMC7010642; doi:10.3389/fphar.2019.01694)
Supplement: Supplementary file 1 [file Image_1.pdf]

A

Meta-regression		Number of obs =	13
REML estimate of between-study variance		tau2 =	0
% residual variation due to heterogeneity		I-squared_res =	0.00%
Proportion of between-study variance explained		Adj R-squared =	100.00%
Joint test for all covariates		Model F(6,6) =	1.45
With Knapp-Hartung modification		Prob > F =	0.3323

logrr	Coef.	Std. Err.	t	P>|t|	[95% Conf.	Interval]
Time	-.072454	.1145169	-0.63	0.550	-.3526667	.2077588
Type	-.0454872	.1138452	-0.40	0.703	-.3240564	.233082
Number	.1644301	.0941716	1.75	0.131	-.0659994	.3948597
L	.0468945	.0626618	0.75	0.483	-.1064335	.2002225
Dose	.1155427	.0997572	1.16	0.291	-.1285543	.3596397
Method	.1102022	.058039	1.90	0.106	-.0318141	.2522184
_cons	-.0032379	.108211	-0.03	0.977	-.2680206	.2615449

B

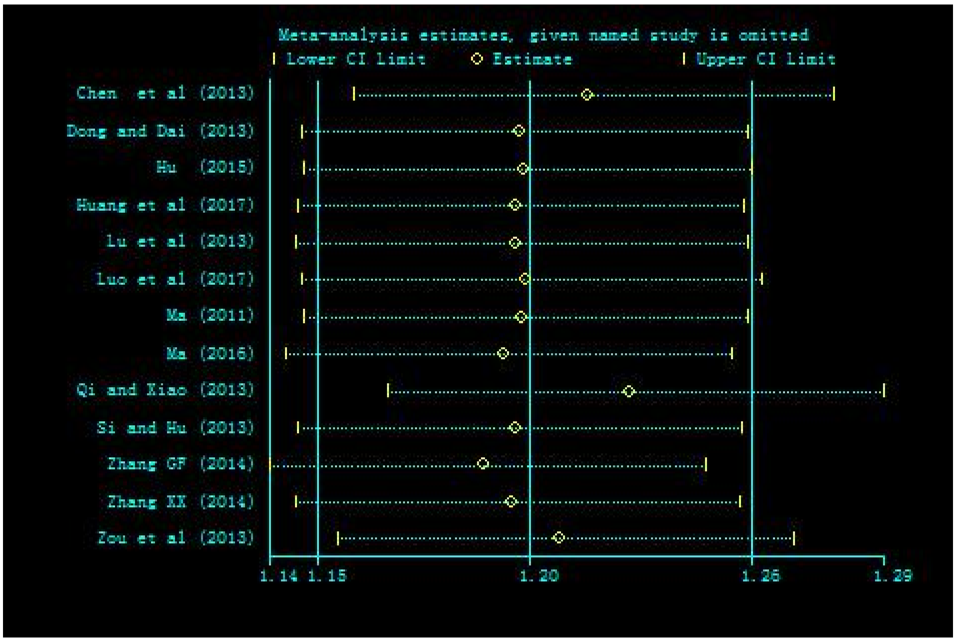

Supplementary figure 1. (A) The meta-regression analysis of berberine-containing quadruple therapy on *H. pylori* eradication rate."L"is the publication year. (B) The sensitivity analysis of berberine-containing quadruple therapy on *H. pylori* eradication rate.
