# Supplementary material for: The Efficacy of Berberine-Containing Quadruple Therapy on Helicobacter Pylori Eradication in China: A Systematic Review and Meta-Analysis of Randomized Clinical Trials
Source: Front Pharmacol. 2020 Feb 4;10:1694. doi: 10.3389/fphar.2019.01694 (PMC7010642; doi:10.3389/fphar.2019.01694)
Supplement: Supplementary file 2 [file Image_2.pdf]

A

Meta-regression	Number of obs = 10
REML estimate of between-study variance	tau2 = 0
% residual variation due to heterogeneity	I-squared_res = 0.00%
Proportion of between-study variance explained	Adj R-squared = 100.00%
Joint test for all covariates	Model F(5,4) = 2.42
With Knapp-Hartung modification	Prob > F = 0.2064

logrr	Coef.	Std. Err.	t	P>|t|	[95% Conf.	Interval]
Time	.1137939	.1102515	1.03	0.360	-.1923133	.4199012
Type	-.139762	.075533	-1.85	0.138	-.3494752	.0699513
Number	-.0386755	.0816893	-0.47	0.661	-.2654812	.1881302
L	.0683854	.0821962	0.83	0.452	-.1598279	.2965988
Dose	.0852291	.0639117	1.33	0.253	-.0922182	.2626765
_cons	.0742196	.0876883	0.85	0.445	-.1692421	.3176812

B

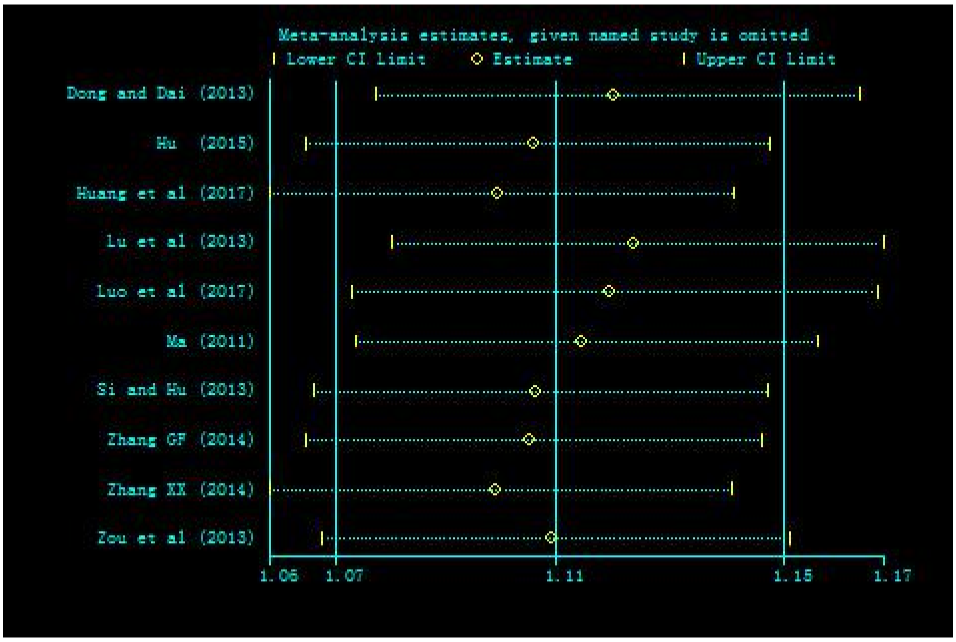

Supplementary figure 2-1. (A) The meta-regression analysis of berberine-containing quadruple therapy on peptic ulcer healing rate."L"is the publication year. (B) The sensitivity analysis of berberine-containing quadruple therapy on peptic ulcer healing rate.
