# Supplementary material for: The Efficacy of Berberine-Containing Quadruple Therapy on Helicobacter Pylori Eradication in China: A Systematic Review and Meta-Analysis of Randomized Clinical Trials
Source: Front Pharmacol. 2020 Feb 4;10:1694. doi: 10.3389/fphar.2019.01694 (PMC7010642; doi:10.3389/fphar.2019.01694)
Supplement: Supplementary file 3 [file Image_3.pdf]

A

Study or Subgroup	Experimental Events	Experimental Total	Control Events	Control Total	Weight	Risk Ratio M-H, Fixed, 95% CI
**2.5.1 120mg**						
Dong 2013	48	50	46	50	9.3%	1.04 [0.94, 1.15]
Lu 2013	53	55	42	45	9.4%	1.03 [0.94, 1.13]
Luo 2017	80	81	74	81	15.0%	1.08 [1.01, 1.16]
Ma 2011	24	25	14	15	3.5%	1.03 [0.88, 1.20]
Zhang XX2014	92	100	74	100	15.0%	1.24 [1.09, 1.42]
**Subtotal (95% CI)**		**311**		**291**	**52.2%**	**1.11 [1.05, 1.17]**
Total events	297		250			
Heterogeneity: Chi² = 8.01, df = 4 (P = 0.09); I² = 50%						
Test for overall effect: Z = 3.94 (P < 0.0001)						
**2.5.2 300mg**						
Huang 2017	93	100	76	100	15.4%	1.22 [1.08, 1.38]
Zou 2013	48	50	43	50	8.7%	1.12 [0.98, 1.27]
**Subtotal (95% CI)**		**150**		**150**	**24.1%**	**1.18 [1.08, 1.30]**
Total events	141		119			
Heterogeneity: Chi² = 1.14, df = 1 (P = 0.29); I² = 12%						
Test for overall effect: Z = 3.66 (P = 0.0003)						
**2.5.3 400mg**						
Si 2013	41	45	34	45	6.9%	1.21 [1.00, 1.46]
**Subtotal (95% CI)**		**45**		**45**	**6.9%**	**1.21 [1.00, 1.46]**
Total events	41		34			
Heterogeneity: Not applicable						
Test for overall effect: Z = 1.94 (P = 0.05)						
**2.5.4 500mg**						
Hu 2015	51	53	44	53	8.9%	1.16 [1.01, 1.32]
Zhang GF2014	47	50	39	50	7.9%	1.21 [1.02, 1.42]
**Subtotal (95% CI)**		**103**		**103**	**16.8%**	**1.18 [1.06, 1.31]**
Total events	98		83			
Heterogeneity: Chi² = 0.13, df = 1 (P = 0.71); I² = 0%						
Test for overall effect: Z = 3.12 (P = 0.002)						
**Total (95% CI)**		**609**		**589**	**100.0%**	**1.15 [1.10, 1.19]**
Total events	577		486			
Heterogeneity: Chi² = 16.02, df = 9 (P = 0.07); I² = 44%						
Test for overall effect: Z = 6.50 (P < 0.00001)						
Test for subgroup differences: Chi² = 2.59, df = 3 (P = 0.46), I² = 0%						

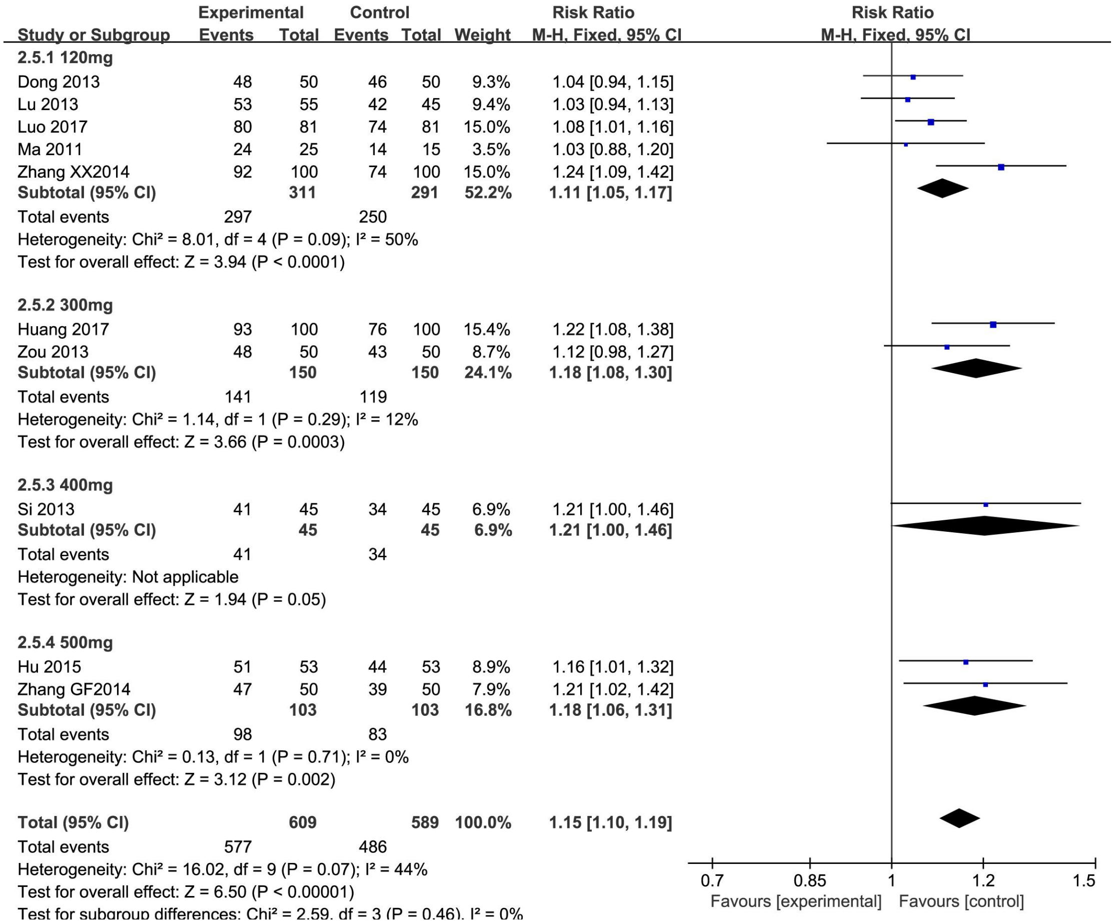

B

Study or Subgroup	Experimental Events	Experimental Total	Control Events	Control Total	Weight	Risk Ratio M-H, Fixed, 95% CI
**2.2.1 RAB**						
Dong 2013	48	50	46	50	9.3%	1.04 [0.94, 1.15]
Lu 2013	53	55	42	45	9.4%	1.03 [0.94, 1.13]
Ma 2011	24	25	14	15	3.5%	1.03 [0.88, 1.20]
Zhang XX2014	92	100	74	100	15.0%	1.24 [1.09, 1.42]
**Subtotal (95% CI)**		**230**		**210**	**37.2%**	**1.12 [1.05, 1.20]**
Total events	217		176			
Heterogeneity: Chi² = 8.45, df = 3 (P = 0.04); I² = 65%						
Test for overall effect: Z = 3.37 (P = 0.0007)						
**2.2.2 OME**						
Hu 2015	51	53	44	53	8.9%	1.16 [1.01, 1.32]
Huang 2017	93	100	76	100	15.4%	1.22 [1.08, 1.38]
Luo 2017	80	81	74	81	15.0%	1.08 [1.01, 1.16]
Si 2013	41	45	34	45	6.9%	1.21 [1.00, 1.46]
Zhang GF2014	47	50	39	50	7.9%	1.21 [1.02, 1.42]
Zou 2013	48	50	43	50	8.7%	1.12 [0.98, 1.27]
**Subtotal (95% CI)**		**379**		**379**	**62.8%**	**1.16 [1.10, 1.22]**
Total events	360		310			
Heterogeneity: Chi² = 5.31, df = 5 (P = 0.38); I² = 6%						
Test for overall effect: Z = 5.59 (P < 0.00001)						
**Total (95% CI)**		**609**		**589**	**100.0%**	**1.15 [1.10, 1.19]**
Total events	577		486			
Heterogeneity: Chi² = 16.02, df = 9 (P = 0.07); I² = 44%						
Test for overall effect: Z = 6.50 (P < 0.00001)						
Test for subgroup differences: Chi² = 0.72, df = 1 (P = 0.40), I² = 0%						

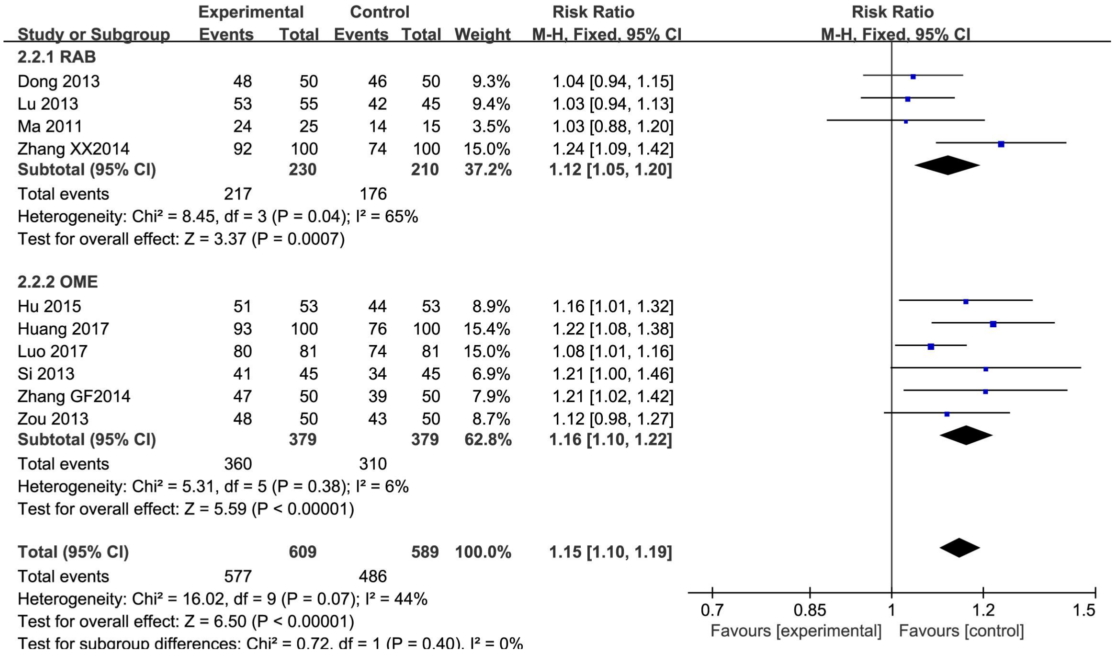

C

Study or Subgroup	Experimental Events	Experimental Total	Control Events	Control Total	Weight	Risk Ratio M-H, Fixed, 95% CI
**2.3.1 ＜200**						
Huang 2017	93	100	76	100	15.4%	1.22 [1.08, 1.38]
Si 2013	41	45	34	45	6.9%	1.21 [1.00, 1.46]
Zou 2013	48	50	43	50	8.7%	1.12 [0.98, 1.27]
**Subtotal (95% CI)**		**195**		**195**	**31.0%**	**1.19 [1.10, 1.29]**
Total events	182		153			
Heterogeneity: Chi² = 1.21, df = 2 (P = 0.55); I² = 0%						
Test for overall effect: Z = 4.13 (P < 0.0001)						
**2.3.2 ≥200**						
Dong 2013	48	50	46	50	9.3%	1.04 [0.94, 1.15]
Hu 2015	51	53	44	53	8.9%	1.16 [1.01, 1.32]
Lu 2013	53	55	42	45	9.4%	1.03 [0.94, 1.13]
Luo 2017	80	81	74	81	15.0%	1.08 [1.01, 1.16]
Ma 2011	24	25	14	15	3.5%	1.03 [0.88, 1.20]
Zhang GF2014	47	50	39	50	7.9%	1.21 [1.02, 1.42]
Zhang XX2014	92	100	74	100	15.0%	1.24 [1.09, 1.42]
**Subtotal (95% CI)**		**414**		**394**	**69.0%**	**1.13 [1.08, 1.18]**
Total events	395		333			
Heterogeneity: Chi² = 11.20, df = 6 (P = 0.08); I² = 46%						
Test for overall effect: Z = 5.02 (P < 0.00001)						
**Total (95% CI)**		**609**		**589**	**100.0%**	**1.15 [1.10, 1.19]**
Total events	577		486			
Heterogeneity: Chi² = 16.02, df = 9 (P = 0.07); I² = 44%						
Test for overall effect: Z = 6.50 (P < 0.00001)						
Test for subgroup differences: Chi² = 1.29, df = 1 (P = 0.26), I² = 22.3%						

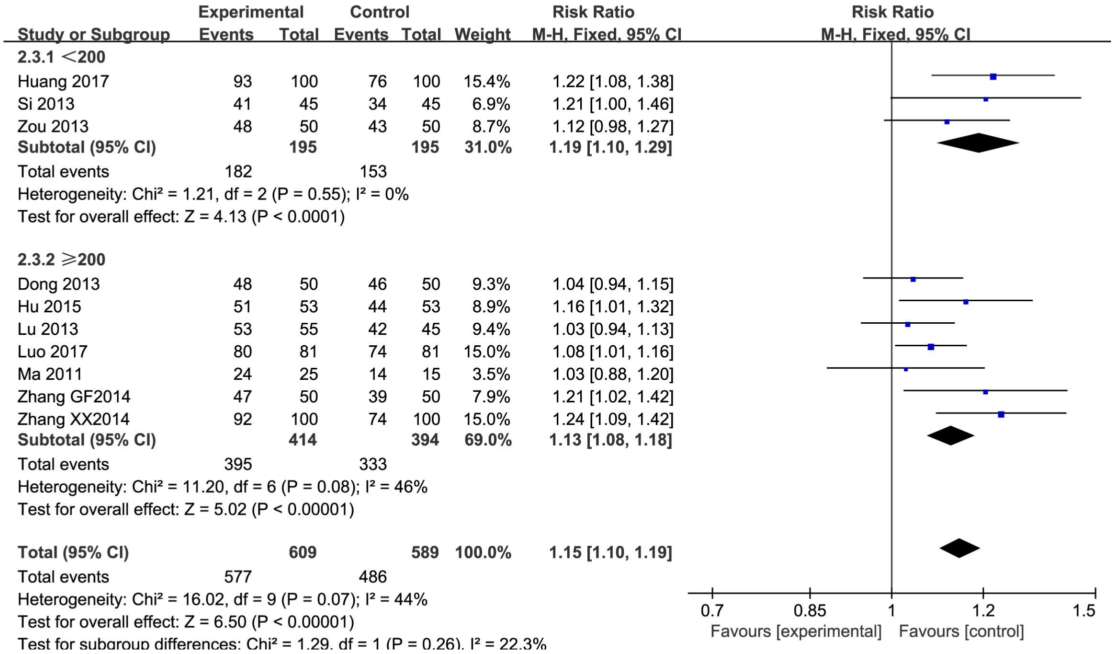

D

Study or Subgroup	Experimental Events	Experimental Total	Control Events	Control Total	Weight	Risk Ratio M-H, Fixed, 95% CI
**2.4.1 2011-2013**						
Dong 2013	48	50	46	50	9.3%	1.04 [0.94, 1.15]
Lu 2013	53	55	42	45	9.4%	1.03 [0.94, 1.13]
Ma 2011	24	25	14	15	3.5%	1.03 [0.88, 1.20]
Si 2013	41	45	34	45	6.9%	1.21 [1.00, 1.46]
Zou 2013	48	50	43	50	8.7%	1.12 [0.98, 1.27]
**Subtotal (95% CI)**		**225**		**205**	**37.8%**	**1.09 [1.02, 1.15]**
Total events	214		179			
Heterogeneity: Chi² = 3.54, df = 4 (P = 0.47); I² = 0%						
Test for overall effect: Z = 2.74 (P = 0.006)						
**2.4.2 2014-2017**						
Hu 2015	51	53	44	53	8.9%	1.16 [1.01, 1.32]
Huang 2017	93	100	76	100	15.4%	1.22 [1.08, 1.38]
Luo 2017	80	81	74	81	15.0%	1.08 [1.01, 1.16]
Zhang GF2014	47	50	39	50	7.9%	1.21 [1.02, 1.42]
Zhang XX2014	92	100	74	100	15.0%	1.24 [1.09, 1.42]
**Subtotal (95% CI)**		**384**		**384**	**62.2%**	**1.18 [1.12, 1.25]**
Total events	363		307			
Heterogeneity: Chi² = 7.09, df = 4 (P = 0.13); I² = 44%						
Test for overall effect: Z = 5.95 (P < 0.00001)						
**Total (95% CI)**		**609**		**589**	**100.0%**	**1.15 [1.10, 1.19]**
Total events	577		486			
Heterogeneity: Chi² = 16.02, df = 9 (P = 0.07); I² = 44%						
Test for overall effect: Z = 6.50 (P < 0.00001)						
Test for subgroup differences: Chi² = 4.29, df = 1 (P = 0.04), I² = 76.7%						

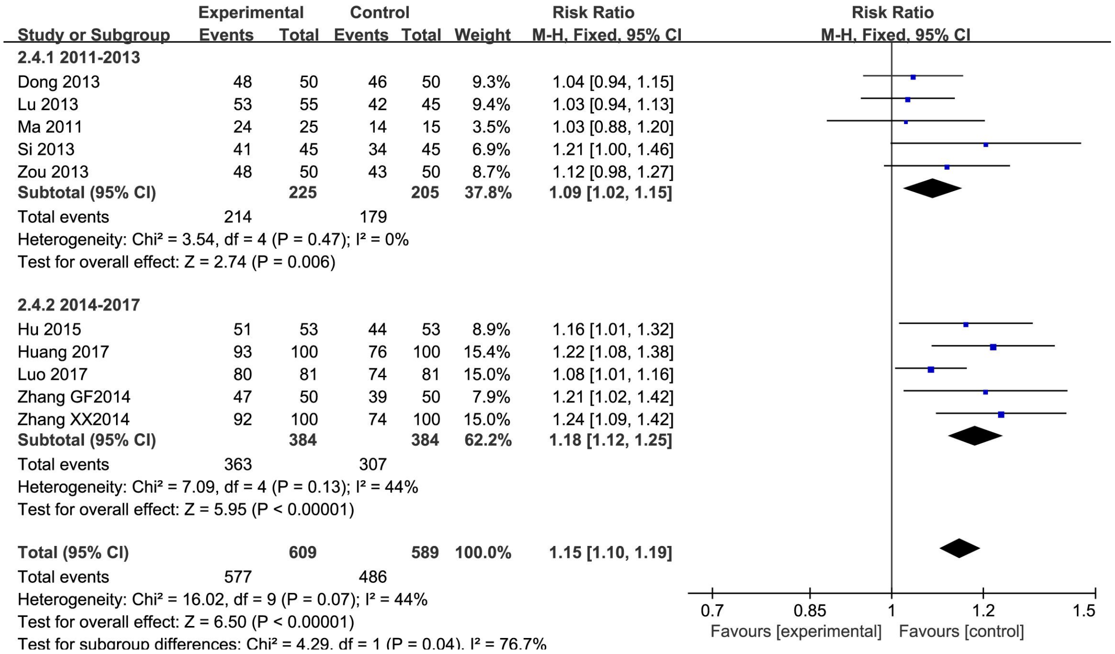

Supplementary figure 2-2. (A) Subgroup analysis of the peptic ulcer healing rate about the dose of berberine. (B) Subgroup analysis of thepeptic ulcer healing rate about the PPI type. (C) Subgroup analysis of the peptic ulcer healing rate about the total number of participants. (D) Subgroup analysis of the peptic ulcer healing rate about the publication year.
