# Supplementary material for: The Efficacy of Berberine-Containing Quadruple Therapy on Helicobacter Pylori Eradication in China: A Systematic Review and Meta-Analysis of Randomized Clinical Trials
Source: Front Pharmacol. 2020 Feb 4;10:1694. doi: 10.3389/fphar.2019.01694 (PMC7010642; doi:10.3389/fphar.2019.01694)
Supplement: Supplementary file 4 [file Image_4.pdf]

A

```
Meta-regression                                      Number of obs   =         5
REML estimate of between-study variance              tau2            =    .01048
% residual variation due to heterogeneity            I-squared_res   =    68.05%
Proportion of between-study variance explained       Adj R-squared   =        .%
Joint test for all covariates                        Model F(2,2)    =      0.08
With Knapp-Hartung modification                      Prob > F        =    0.9256
```

logrr	Coef.	Std. Err.	t	P>|t|	[95% Conf.	Interval]
Time	-.0559713	.1402978	-0.40	0.728	-.659624	.5476814
Dose	.0383715	.1390125	0.28	0.808	-.5597511	.6364941
_cons	.1339328	.0879002	1.52	0.267	-.2442714	.512137

```
Meta-regression                                      Number of obs   =         5
REML estimate of between-study variance              tau2            =   .003841
% residual variation due to heterogeneity            I-squared_res   =    52.67%
Proportion of between-study variance explained       Adj R-squared   =        .%
With Knapp-Hartung modification
```

logrr	Coef.	Std. Err.	t	P>|t|	[95% Conf.	Interval]
Type	-.0248278	.0877186	-0.28	0.796	-.3039877	.2543321
_cons	.1203505	.0709712	1.70	0.188	-.1055114	.3462124

B

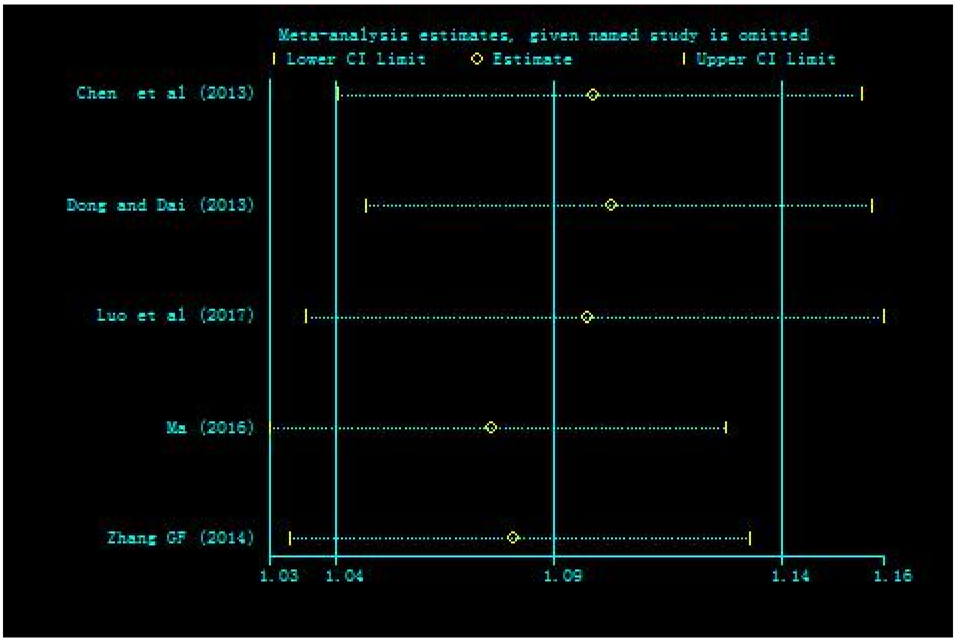

Supplementary figure 3-1. (A) The meta-regression analyse of berberine-containing quadruple therapy on relieving rate of clinical symptoms. (B) The sensitivity analysis of berberine-containing quadruple therapy on relieving rate of clinical symptoms.
