# Supplementary material for: The Efficacy of Berberine-Containing Quadruple Therapy on Helicobacter Pylori Eradication in China: A Systematic Review and Meta-Analysis of Randomized Clinical Trials
Source: Front Pharmacol. 2020 Feb 4;10:1694. doi: 10.3389/fphar.2019.01694 (PMC7010642; doi:10.3389/fphar.2019.01694)
Supplement: Supplementary file 5 [file Image_5.pdf]

A

| Study or Subgroup | Experimental Events | Experimental Total | Control Events | Control Total | Weight | Risk Ratio M-H, Fixed, 95% CI |
|---|---|---|---|---|---|---|
| **3.2.1 RAB** | | | | | | |
| Dong 2013 | 48 | 50 | 46 | 50 | 14.7% | 1.04 [0.94, 1.15] |
| Ma 2016 | 52 | 55 | 40 | 55 | 12.8% | 1.30 [1.09, 1.55] |
| **Subtotal (95% CI)** | | **105** | | **105** | **27.6%** | **1.16 [1.05, 1.28]** |
| Total events | 100 | | 86 | | | |
| Heterogeneity: Chi² = 6.14, df = 1 (P = 0.01); I² = 84% | | | | | | |
| Test for overall effect: Z = 2.98 (P = 0.003) | | | | | | |
| **3.2.2 OME** | | | | | | |
| Chen 2013 | 128 | 140 | 107 | 125 | 36.2% | 1.07 [0.98, 1.17] |
| Luo 2017 | 80 | 81 | 74 | 81 | 23.7% | 1.08 [1.01, 1.16] |
| Zhang GF2014 | 47 | 50 | 39 | 50 | 12.5% | 1.21 [1.02, 1.42] |
| **Subtotal (95% CI)** | | **271** | | **256** | **72.4%** | **1.10 [1.03, 1.16]** |
| Total events | 255 | | 220 | | | |
| Heterogeneity: Chi² = 1.77, df = 2 (P = 0.41); I² = 0% | | | | | | |
| Test for overall effect: Z = 3.13 (P = 0.002) | | | | | | |
| **Total (95% CI)** | | **376** | | **361** | **100.0%** | **1.11 [1.06, 1.17]** |
| Total events | 355 | | 306 | | | |
| Heterogeneity: Chi² = 7.17, df = 4 (P = 0.13); I² = 44% | | | | | | |
| Test for overall effect: Z = 4.26 (P < 0.0001) | | | | | | |
| Test for subgroup differences: Chi² = 1.02, df = 1 (P = 0.31), I² = 2.3% | | | | | | |

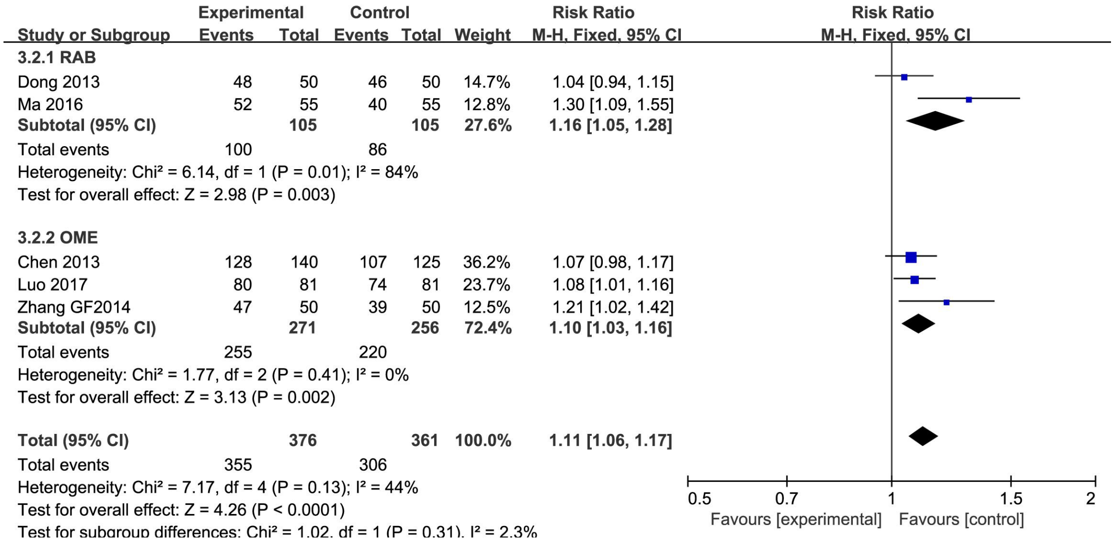


B

| Study or Subgroup | Experimental Events | Experimental Total | Control Events | Control Total | Weight | Risk Ratio M-H, Fixed, 95% CI |
|---|---|---|---|---|---|---|
| **3.1.1 7days** | | | | | | |
| Dong 2013 | 48 | 50 | 46 | 50 | 14.7% | 1.04 [0.94, 1.15] |
| Ma 2016 | 52 | 55 | 40 | 55 | 12.8% | 1.30 [1.09, 1.55] |
| **Subtotal (95% CI)** | | **105** | | **105** | **27.6%** | **1.16 [1.05, 1.28]** |
| Total events | 100 | | 86 | | | |
| Heterogeneity: Chi² = 6.14, df = 1 (P = 0.01); I² = 84% | | | | | | |
| Test for overall effect: Z = 2.98 (P = 0.003) | | | | | | |
| **3.1.2 14days** | | | | | | |
| Chen 2013 | 128 | 140 | 107 | 125 | 36.2% | 1.07 [0.98, 1.17] |
| Luo 2017 | 80 | 81 | 74 | 81 | 23.7% | 1.08 [1.01, 1.16] |
| Zhang GF2014 | 47 | 50 | 39 | 50 | 12.5% | 1.21 [1.02, 1.42] |
| **Subtotal (95% CI)** | | **271** | | **256** | **72.4%** | **1.10 [1.03, 1.16]** |
| Total events | 255 | | 220 | | | |
| Heterogeneity: Chi² = 1.77, df = 2 (P = 0.41); I² = 0% | | | | | | |
| Test for overall effect: Z = 3.13 (P = 0.002) | | | | | | |
| **Total (95% CI)** | | **376** | | **361** | **100.0%** | **1.11 [1.06, 1.17]** |
| Total events | 355 | | 306 | | | |
| Heterogeneity: Chi² = 7.17, df = 4 (P = 0.13); I² = 44% | | | | | | |
| Test for overall effect: Z = 4.26 (P < 0.0001) | | | | | | |
| Test for subgroup differences: Chi² = 1.02, df = 1 (P = 0.31), I² = 2.3% | | | | | | |

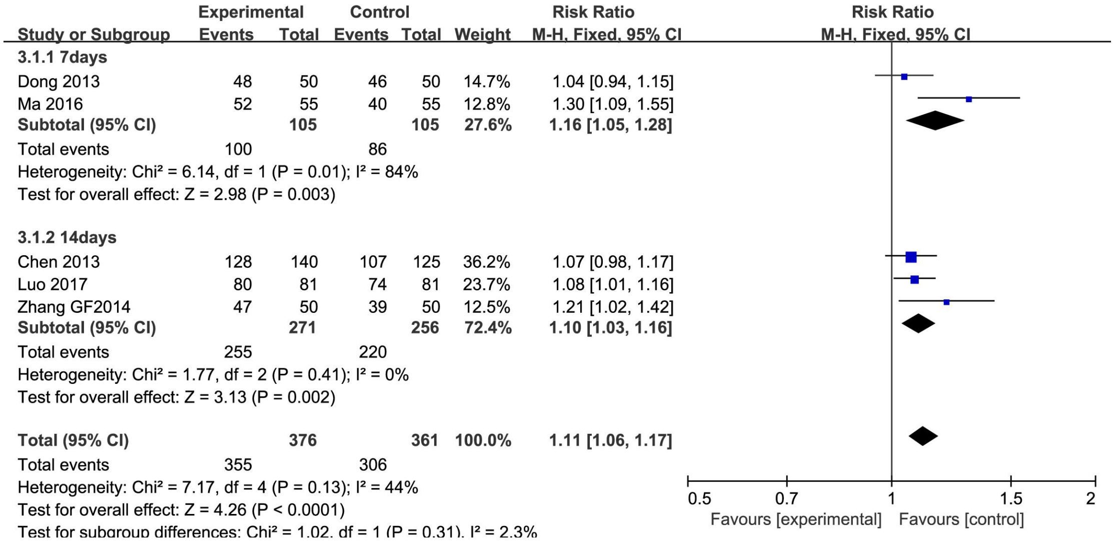

C

| Study or Subgroup | Experimental Events | Experimental Total | Control Events | Control Total | Weight | Risk Ratio M-H, Fixed, 95% CI |
|---|---|---|---|---|---|---|
| **3.3.1 120mg** | | | | | | |
| Dong 2013 | 48 | 50 | 46 | 50 | 14.7% | 1.04 [0.94, 1.15] |
| Luo 2017 | 80 | 81 | 74 | 81 | 23.7% | 1.08 [1.01, 1.16] |
| Ma 2016 | 52 | 55 | 40 | 55 | 12.8% | 1.30 [1.09, 1.55] |
| **Subtotal (95% CI)** | | **186** | | **186** | **51.3%** | **1.13 [1.06, 1.20]** |
| Total events | 180 | | 160 | | | |
| Heterogeneity: Chi² = 6.06, df = 2 (P = 0.05); I² = 67% | | | | | | |
| Test for overall effect: Z = 3.66 (P = 0.0003) | | | | | | |
| **3.3.2 300mg** | | | | | | |
| Chen 2013 | 128 | 140 | 107 | 125 | 36.2% | 1.07 [0.98, 1.17] |
| **Subtotal (95% CI)** | | **140** | | **125** | **36.2%** | **1.07 [0.98, 1.17]** |
| Total events | 128 | | 107 | | | |
| Heterogeneity: Not applicable | | | | | | |
| Test for overall effect: Z = 1.47 (P = 0.14) | | | | | | |
| **3.3.3 500mg** | | | | | | |
| Zhang GF2014 | 47 | 50 | 39 | 50 | 12.5% | 1.21 [1.02, 1.42] |
| **Subtotal (95% CI)** | | **50** | | **50** | **12.5%** | **1.21 [1.02, 1.42]** |
| Total events | 47 | | 39 | | | |
| Heterogeneity: Not applicable | | | | | | |
| Test for overall effect: Z = 2.24 (P = 0.02) | | | | | | |
| **Total (95% CI)** | | **376** | | **361** | **100.0%** | **1.11 [1.06, 1.17]** |
| Total events | 355 | | 306 | | | |
| Heterogeneity: Chi² = 7.17, df = 4 (P = 0.13); I² = 44% | | | | | | |
| Test for overall effect: Z = 4.26 (P < 0.0001) | | | | | | |
| Test for subgroup differences: Chi² = 1.87, df = 2 (P = 0.39), I² = 0% | | | | | | |

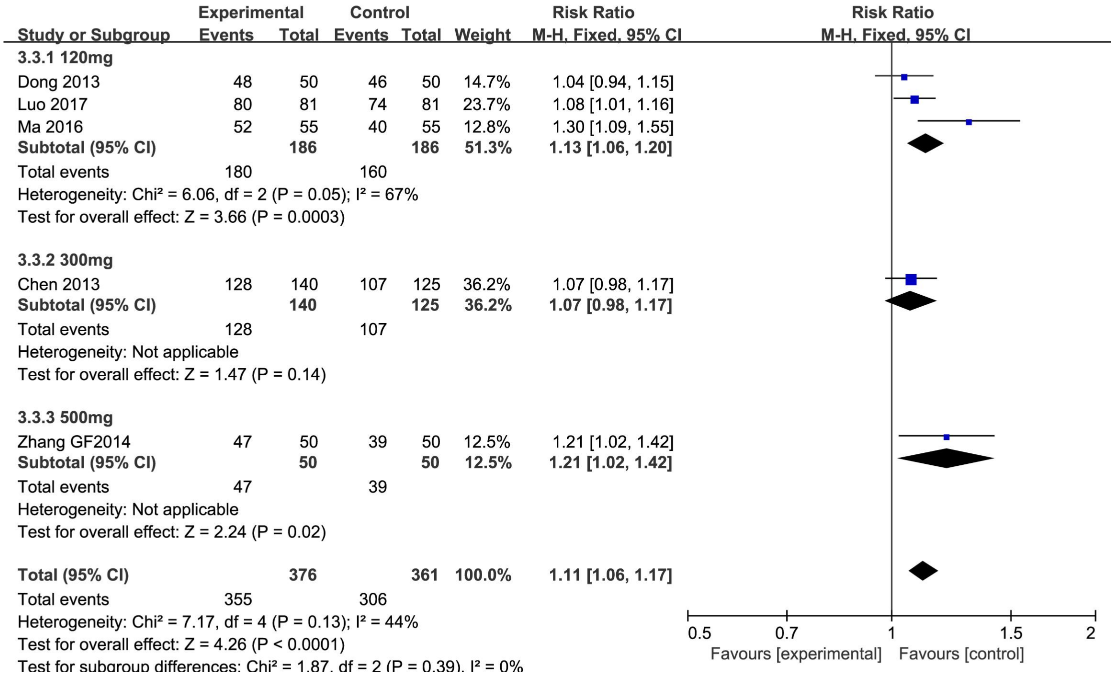


Supplementary figure 3-2. (A) Subgroup analysis of the relieving rate of clinical symptoms about the PPI type. (B) Subgroup analysis of the relieving rate of clinical symptoms about the time of treatment duration. (C) Subgroup analysis of the relieving rate of clinical symptoms about the dose of berberine.
