# Supplementary material for: The Efficacy of Berberine-Containing Quadruple Therapy on Helicobacter Pylori Eradication in China: A Systematic Review and Meta-Analysis of Randomized Clinical Trials
Source: Front Pharmacol. 2020 Feb 4;10:1694. doi: 10.3389/fphar.2019.01694 (PMC7010642; doi:10.3389/fphar.2019.01694)
Supplement: Supplementary file 6 [file Image_6.pdf]

A

```
Meta-regression                                    Number of obs  =        13
REML estimate of between-study variance            tau2           =         0
% residual variation due to heterogeneity          I-squared_res  =     0.00%
Proportion of between-study variance explained     Adj R-squared  =   100.00%
Joint test for all covariates                      Model F(5,7)   =      4.64
With Knapp-Hartung modification                    Prob > F       =    0.0346
```

logrr	Coef.	Std. Err.	t	P>|t|	[95% Conf.	Interval]
Time	-.5293625	.3457144	-1.53	0.170	-1.346847	.2881223
Type	.5526185	.3429245	1.61	0.151	-.2582691	1.363506
Number	.2441416	.2452077	1.00	0.353	-.3356825	.8239658
L	-.4627358	.2379482	-1.94	0.093	-1.025394	.0999223
Dose	.4276388	.2756111	1.55	0.165	-.2240779	1.079356
_cons	-.6065891	.2549947	-2.38	0.049	-1.209556	-.0036223

B

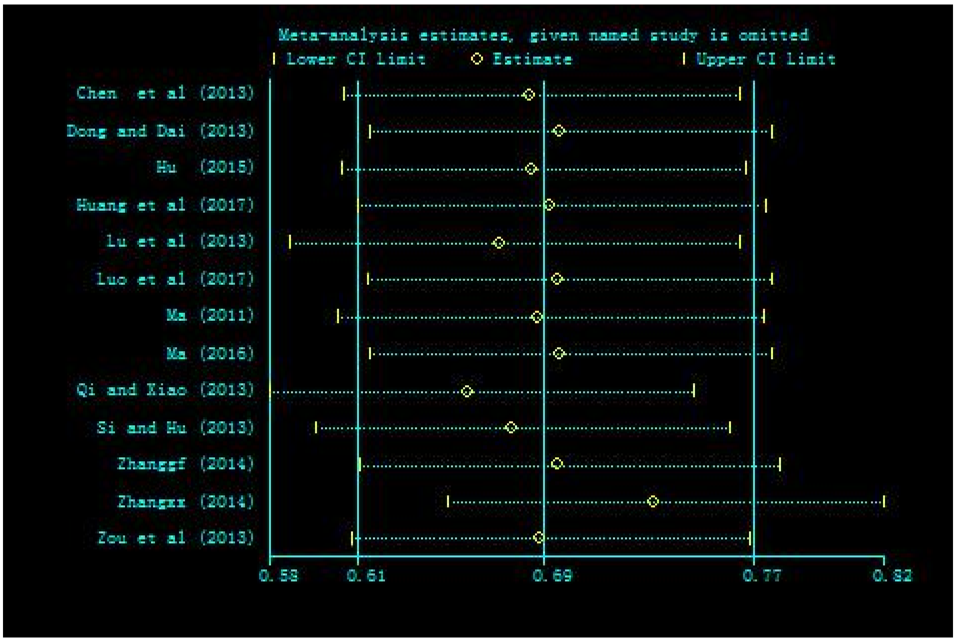

Supplementary figure 4-1. (A) The meta-regression analyse of berberine-containing quadruple therapy on adverse effect."L"is the publication year. (B) The sensitivity analysis of berberine-containing quadruple therapy on adverse events.
