# Supplementary material for: The Efficacy of Berberine-Containing Quadruple Therapy on Helicobacter Pylori Eradication in China: A Systematic Review and Meta-Analysis of Randomized Clinical Trials
Source: Front Pharmacol. 2020 Feb 4;10:1694. doi: 10.3389/fphar.2019.01694 (PMC7010642; doi:10.3389/fphar.2019.01694)
Supplement: Supplementary file 7 [file Image_7.pdf]

A

Study or Subgroup	Experimental Events	Experimental Total	Control Events	Control Total	Weight	Risk Ratio M-H, Random, 95% CI
**1.11.1 RAB**						
Dong 2013	5	50	13	50	3.7%	0.38 [0.15, 1.00]
Lu 2013	47	84	59	78	13.7%	0.74 [0.59, 0.93]
Ma 2011	28	54	31	42	11.9%	0.70 [0.51, 0.96]
Ma 2016	2	55	12	55	1.9%	0.17 [0.04, 0.71]
Qi 2013	67	210	81	210	13.0%	0.83 [0.64, 1.07]
Zhang XX2014	16	100	62	100	8.9%	0.26 [0.16, 0.41]
**Subtotal (95% CI)**		**553**		**535**	**53.1%**	**0.54 [0.37, 0.79]**
Total events	165		258			
Heterogeneity: Tau² = 0.15; Chi² = 25.66, df = 5 (P = 0.0001); I² = 81%						
Test for overall effect: Z = 3.21 (P = 0.001)						
**1.11.2 OME**						
Chen 2013	10	140	8	125	4.1%	1.12 [0.45, 2.74]
Hu 2015	14	53	17	53	7.0%	0.82 [0.45, 1.49]
Huang 2017	18	100	28	100	8.1%	0.64 [0.38, 1.08]
Luo 2017	5	81	13	81	3.6%	0.38 [0.14, 1.03]
Si 2013	24	45	30	45	11.4%	0.80 [0.57, 1.13]
Zhang GF2014	21	50	33	50	10.6%	0.64 [0.43, 0.93]
Zou 2013	4	50	4	50	2.2%	1.00 [0.26, 3.78]
**Subtotal (95% CI)**		**519**		**504**	**46.9%**	**0.72 [0.59, 0.88]**
Total events	96		133			
Heterogeneity: Tau² = 0.00; Chi² = 3.86, df = 6 (P = 0.70); I² = 0%						
Test for overall effect: Z = 3.16 (P = 0.002)						
**Total (95% CI)**		**1072**		**1039**	**100.0%**	**0.64 [0.52, 0.79]**
Total events	261		391			
Heterogeneity: Tau² = 0.07; Chi² = 29.20, df = 12 (P = 0.004); I² = 59%						
Test for overall effect: Z = 4.19 (P < 0.0001)						
Test for subgroup differences: Chi² = 1.83, df = 1 (P = 0.18), I² = 45.3%						

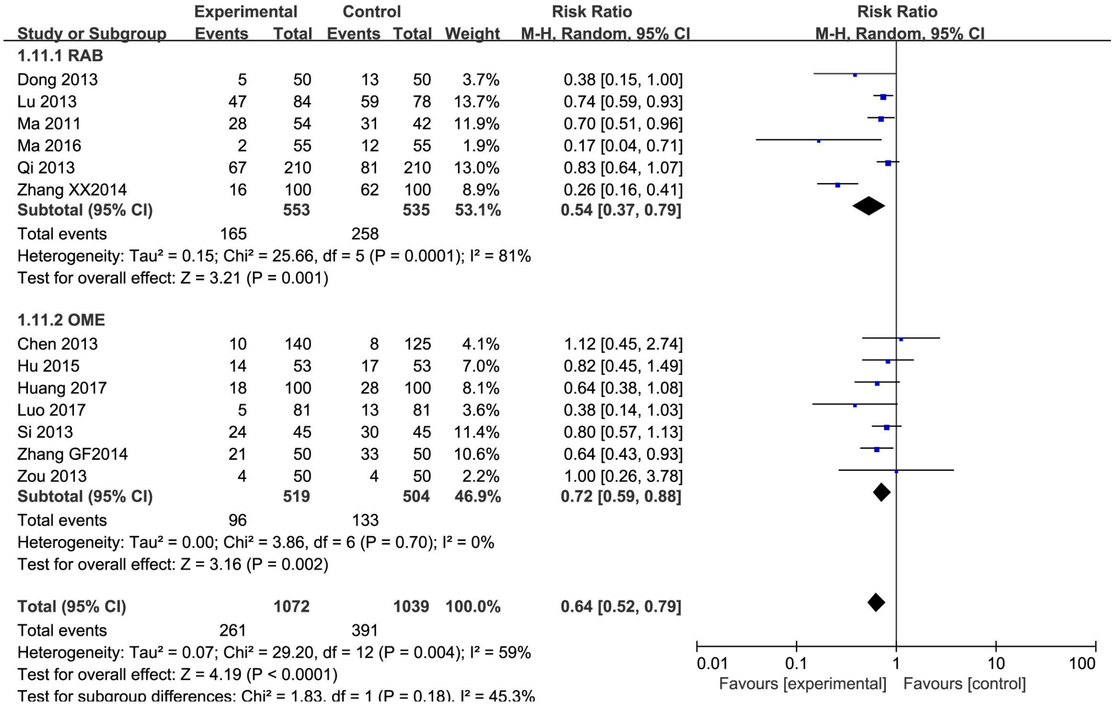

B

Study or Subgroup	Experimental Events	Experimental Total	Control Events	Control Total	Weight	Risk Ratio M-H, Random, 95% CI
**1.13.1 2011-2013**						
Chen 2013	10	140	8	125	4.1%	1.12 [0.45, 2.74]
Dong 2013	5	50	13	50	3.7%	0.38 [0.15, 1.00]
Lu 2013	47	84	59	78	13.7%	0.74 [0.59, 0.93]
Ma 2011	28	54	31	42	11.9%	0.70 [0.51, 0.96]
Qi 2013	67	210	81	210	13.0%	0.83 [0.64, 1.07]
Si 2013	24	45	30	45	11.4%	0.80 [0.57, 1.13]
Zou 2013	4	50	4	50	2.2%	1.00 [0.26, 3.78]
**Subtotal (95% CI)**		**633**		**600**	**60.0%**	**0.76 [0.67, 0.87]**
Total events	185		226			
Heterogeneity: Tau² = 0.00; Chi² = 3.63, df = 6 (P = 0.73); I² = 0%						
Test for overall effect: Z = 3.94 (P < 0.0001)						
**1.13.2 2014-2017**						
Hu 2015	14	53	17	53	7.0%	0.82 [0.45, 1.49]
Huang 2017	18	100	28	100	8.1%	0.64 [0.38, 1.08]
Luo 2017	5	81	13	81	3.6%	0.38 [0.14, 1.03]
Ma 2016	2	55	12	55	1.9%	0.17 [0.04, 0.71]
Zhang GF2014	21	50	33	50	10.6%	0.64 [0.43, 0.93]
Zhang XX2014	16	100	62	100	8.9%	0.26 [0.16, 0.41]
**Subtotal (95% CI)**		**439**		**439**	**40.0%**	**0.48 [0.30, 0.74]**
Total events	76		165			
Heterogeneity: Tau² = 0.19; Chi² = 15.71, df = 5 (P = 0.008); I² = 68%						
Test for overall effect: Z = 3.27 (P = 0.001)						
**Total (95% CI)**		**1072**		**1039**	**100.0%**	**0.64 [0.52, 0.79]**
Total events	261		391			
Heterogeneity: Tau² = 0.07; Chi² = 29.20, df = 12 (P = 0.004); I² = 59%						
Test for overall effect: Z = 4.19 (P < 0.0001)						
Test for subgroup differences: Chi² = 3.97, df = 1 (P = 0.05), I² = 74.8%						

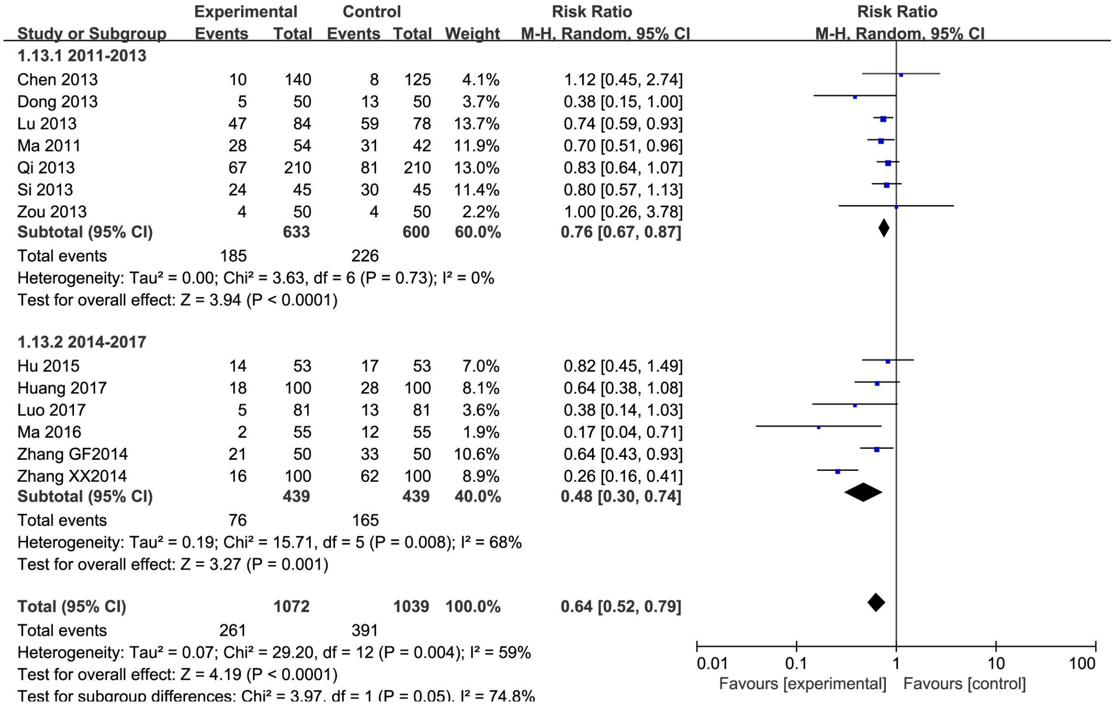

Supplementary figure 4-2. (A) Subgroup analysis of adverse events about the PPI type. (B) Subgroup analysis of adverse events about the publication year.
