# Supplementary material for: The Efficacy of Berberine-Containing Quadruple Therapy on Helicobacter Pylori Eradication in China: A Systematic Review and Meta-Analysis of Randomized Clinical Trials
Source: Front Pharmacol. 2020 Feb 4;10:1694. doi: 10.3389/fphar.2019.01694 (PMC7010642; doi:10.3389/fphar.2019.01694)
Supplement: Supplementary file 8 [file Table_1.pdf]

Table S1. Jadad scores of included studies

Author	Randomized method	Blind	Withdrawals and Dropouts	Total
Ma et al 2011	1	1	1	3
Si and Hu 2013	1	1	1	3
Qi and Xiao 2013	1	1	1	3
Lu et al 2013	1	1	1	3
Zou et al 2013	1	1	1	3
Chen et al 2013	2	1	1	4
Dong and Dai 2013	1	1	1	3
Zhang XX 2014	1	1	1	3
Zhang GF 2014	2	1	1	4
Hu 2015	2	1	1	4
Ma 2016	2	1	1	4
Huang et al 2017	2	1	1	4
Luo et al 2017	1	1	1	3
